# Roles of GalNAc-disialyl Lactotetraosyl Antigens in Renal Cancer Cells

**DOI:** 10.1038/s41598-018-25521-6

**Published:** 2018-05-04

**Authors:** Akiko Tsuchida, Motohiro Senda, Akihiro Ito, Seiichi Saito, Makoto Kiso, Takayuki Ando, Anne Harduin-Lepers, Akio Matsuda, Keiko Furukawa, Koichi Furukawa

**Affiliations:** 1 Laboratory of Glyco-Bioengineering, The Noguchi Institute, Itabashi, 173-0003 Japan; 2 Department of Biochemistry II, Nagoya University Graduate School of Medicine, Nagoya, 466-8550 Japan; 3 Department of Urology, Nagoya University School of Medicine, Nagoya, 466-8550 Japan; 4 Department of Urology, Tohoku University School of Medicine, Sendai, 980-8574 Japan; 5 Department of Urology, University of Ryukyus School of Medicine, Nishihara-cho, 903-0215 Okinawa Japan; 6 Facalty of Applied Biological Sciences, Gifu University, Gifu, 501-1193 Japan; 7 Department of Drug and Food Science, Shizuoka Institute of Environment and Hygiene, Shizuoka, 420-8637 Japan; 8 Unité de Glycobiologie Structurale et Fonctionnelle, Université Lille Nord de France, Villeneuve d’Ascq, 59655 France; 9 Department of Biomedical Sciences, Chubu University College of Life and Health Sciences, Kasugai, 487-8501 Japan; 10 Department of Lifelong Sports and Health Sciences, Chubu University College of Life and Health Sciences, Kasugai, 487-8501 Japan

## Abstract

GalNAc-disialyl Lc4 (GalNAc-DSLc4) was reported as a novel antigen that associated with malignant features of renal cell cancers (RCCs). To clarify roles of GalNAc-DSLc4 in malignant properties of RCCs, we identified B4GalNAc-T2 as a responsible gene for the synthesis of GalNAc-DSLc4, and prepared stable transfectants of GalNAc-T2 cDNA using VMRC-RCW cells, resulting in the establishment of high expressants of GalNAc-DSLc4. They showed increased proliferation and invasion, and specific adhesion to laminin. In the transfectants, PI3K/Akt signals were highly activated by serum stimulation or adhesion to laminin. GalNAc-DSLc4 was co-localized in lipid rafts with integrin β1 and caveolin-1 in both immunoblotting of fractionated detergent extracts and immunocytostaining, particularly when stimulated with serum. Masking of GalNAc-DSLc4 with antibodies as well as PI3K inhibitor suppressed malignant properties of the transfectants. These results suggested that GalNAc-DSLc4 is involved in malignant properties of RCCs by forming a molecular complex with integrins in lipid rafts.

## Introduction

Recently, a number of studies indicate that some aberrant glycosylation is a result of initial oncogenic transformation, playing a key role in the enhancement of invasion and metastasis. It has been reported that high expression of some glyco-epitopes promotes tumor invasion and metastasis, leading to 5–10 year shorter survival rates of patients, whereas expression of some other glyco-epitopes suppresses tumor progression, leading to longer postoperative survival terms^1,2^. Mechanisms for the expression of these novel glyco-epitopes via the activation of respective glycosyltransferase genes have been extensively studied. However, little is understood about mechanisms through which specific glyco-epitopes induce invasive and metastatic phenotypes of tumor cells.

In the case of glycosphingolipids, disialyl glycosphingolipids such as GD3 and GD2 have been reported to be associated with malignant transformation, cancer invasion, metastasis and prognosis^3–6^. Interaction of these disialyl structures with members of a lectin family, siglecs (ssialic acid-binding, immunoglobulin-like lectins), might be considered to be involved in the survival of cancer cells^7,8^.

On the other hand, we have analyzed the mechanism for the synthesis of disialyl ganglioside with α-structure, and isolated cDNAs for the responsible synthetic enzymes, such as ST6GalNAc-V^9^ and ST6GalNAc-VI^10^. We have also determined that ST6GalNAc-VI is the sialyltransferase responsible for the synthesis of disialyl Lewis a (Le^a^), which contains a branched-type disialyl structure on a lacto-core structure^11^.

Interestingly, in addition to disialyl galactosylgloboside (DSGG) identified as one of major disialyl gangliosides from renal cell carcinoma (RCC) tissues^12^, another RCC-specific disialyl ganglioside was found in TOS-1 cell line^13^. This disialyl ganglioside was characterized to have a novel hybrid structure between ganglio-series GM2 and a lacto-series type 1-core. The antigen is termed GalNAc-disialyl Lc4 Cer (IV^4^GalNAcIV^3^NeuAcIII^6^NeuAcLc4; abbreviated GalNAc-DSLc4). Among RCCs, TOS-1 cells were observed to most strongly adhere to lung tissue sections, then, GalNAc-DSLc4 was expected to be a marker indicating possible activity to promote distant metastasis of RCC. ST6GalNAc-VI was also expected to be involved in the synthesis of this novel disialyl ganglioside, GalNAc-DSLc4.

In this study, we identified the responsible transferase for biosynthesis of GalNAc-DSLc4 in RCCs to investigate roles of GalNAc-DSLc4. Then, we established GalNAc-DSLc4-overexpressing transfectant cells from an RCC cell line VMRC-RCW by using cloned B4GalNAc-T2 cDNA^14^, and studied molecular mechanisms for GalNAc-DSLc4-mediated biosignals. We demonstrate here that signaling pathway such as PI3K/Akt undergoes stronger phosphorylation after serum treatment in GalNAc-DSLc4-expressing cells than in control cells, and that GalNAc-DSLc4 is involved in recruitment of integrin β1 into glycolipid-enriched microdomain (GEM)/rafts on the cell surface. GalNAc-DSLc4 actually cooperates with integrin β1 to enhance cell proliferation, invasion, and adhesion to laminin, leading to the increased malignant properties of RCCs.

## Results

### Typing of renal cancer cell lines

**Figure 1:**
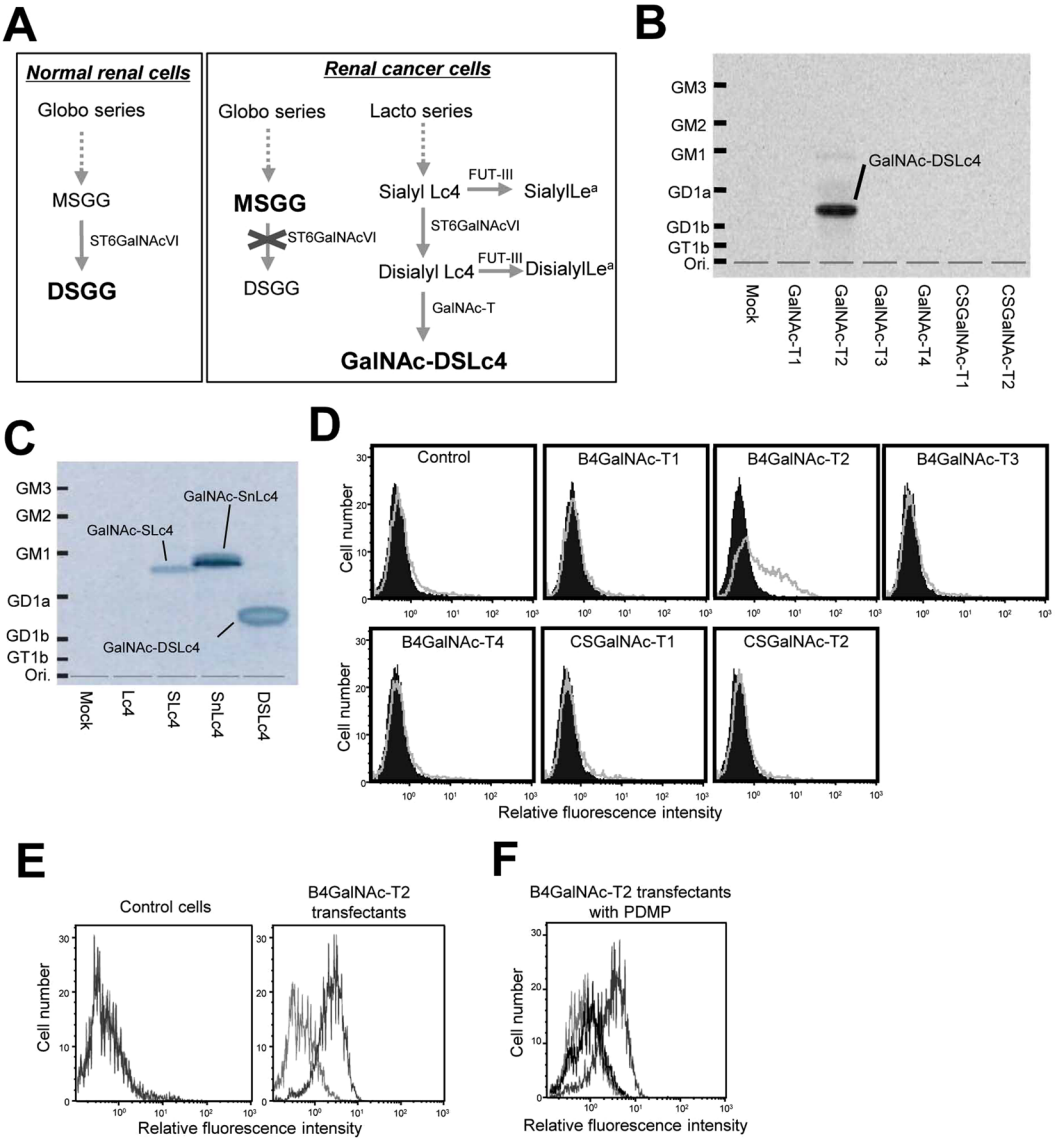
Synthetic pathway of renal cancer-related glycolipids, identification of B4GalNAc-T2 as a B4GalNAc-T responsible for the synthesis of GalNAc-DSLc4, and establishment of stable transfectants with surface expression of GalNAc-DSLc4. (**A**) Synthetic pathway of cancer related-glycolipids in normal renal cells or renal cancer cells. (**B**) TLC of the products from B4GalNAc-T2 enzyme assay. The *N*-acetylgalactosaminyltransferase assay was performed using the membrane fraction with DSLc4 as an acceptor substrate, and the products were analyzed by TLC and auto fluorography. The B4GalNAc-T2 is responsible for synthesis of GalNAc-DSLc4 using DSLc4 as an acceptor. (**C**) Substrate specificity of B4GalNAc-T2 transferase for lacto- or neolacto-series glycolipids. The *N*-acetylgalactosaminyltransferase assay was performed using lacto- and neolacto-series acceptors (Lc4, SLc4, SnLc4, and DSLc4). (**D**) Flow cytometric patterns of a RCC line (TUHR14TKB) transfected with individual cDNAs indicated. (**E**) Establishment of stable transfectants from VMRC-RCW cells. Surface expression of GalNAc-DSLc4 antigens in the transfectants by flow cytometric analysis using RM2 antibody. *Gray* profiles mean RM2-stained cells and *light gray* profiles mean negative cells. (**F**) Reduction of GalNAc-DSLc4 expression by D-PDMP treatment (for 6 days, conc. at 50 μM). *Black* profiles mean reduced RM2 cells in flow cytometric assay.

Expression of globo-series and lacto-series glycosphingolipids in 20 renal cancer cell lines and normal HRPTE cells were analyzed by flow cytometry (Table 1). It was revealed that high expression of monosialyl galactosylgloboside (MSGG) was detected in almost all RCC lines, whereas DSGG expression was minimal or none in the RCC lines as shown previously^15^. In turn, high expression levels of DSGG and low expression levels of MSGG were detected in the normal human renal proximal tubule epithelial cells. Thus, RCC lines generally showed high expression of globo-series glycolipids and low expression of lacto-series glycolipids. But increased expression of a rare lacto-series glycolipid GalNAc-DSLc4 was found in majority of RCC lines (Fig. 1A).

**Table 1:** Expression pattern of renal cancer-related glycolipids.

cell line	Globo series		Lacto series	
	MSGG	DSGG	DSLc4	GalNAc DSLc4
SK-RC 1	+++	—	—	+++
SK-RC 6	+++	++	+	++
SK-RC 7	+	+	++	++
SK-RC 17	+++	±	—	+
SK-RC 29	+++	—	—	—
SK-RC 35	+++	—	—	—
SK-RC 39	+++	—	—	+
SK-RC 44	—	—	—	+
SK-RC 45	+	—	—	—
SK-RC 99	+++	+	±	+
Moroff	—	±	+++	+
OS-RC-2	+++	—	—	+++
Caki-1	+++	—	—	—
VMRC-RCW	+++	+	++	++
VMRC-RCZ	—	—	—	—
RCC10RGB	+++	—	++	+++
TUHR4TKB	+++	—	—	+++
TUHR10TKB	+++	—	—	+++
TUHR14TKB	—	—	+++	+
ACHN	+++	++	—	—
HRPTE	+	+++	—	—

Cell surface expression of globo- and lacto-series glycolipids in 20 renal cancer cell lines and HRPTE (human renal proximal tubule epithelial cells) were analyzed by flow cytometry using anti-glycolipid mAbs as described in “Materials and Methods”. The glycolipid expression levels were classified into 5 groups based on the percentages of positive cells. +++, 70–100%; ++, 40–70%; +, 10–40%; ±, 5–10%; —, 0–5%.

### Identification of B4GalNAc-T2 as a responsible enzyme for the synthesis of GalNAc-DSLc4

To identify the B4GalNAc-T responsible for the synthesis of GalNAc-DSLc4, we prepared expression vectors for 6 B4GalNAc-Ts and examined the enzyme activity to synthesize GalNAc-DSLc4 from DSLc4 as an acceptor using the extracts from cells transfected with B4GalNAc-T1, B4GalNAc-T2, B4GalNAc-T3, B4GalNAc-T4, CSGalNAc-T1, or CSGalNAc-T2 expression vectors. The results showed that B4GalNAc-T2 generated one new band at the migration site of GalNAc-DSLc4 (Fig. 1B). Substrate specificity of B4GalNAc-T2 was investigated using various lacto-series glycolipids as acceptors (Fig. 1C). The result showed that B4GalNAc-T2 transfers GalNAc onto the position C4 of Gal in Neu5Acα2,3-Gal- structure with β linkage. To investigate whether GalNAc-DSLc4 is synthesized *in vivo*, expression vectors encoding B4GalNAc-Ts were transfected into THUR14TKB cells, in which a large amount of the precursor (DSLc4) exists. The results of flow cytometric analysis are shown (Fig. 1D). Only B4GalNAc-T2 could induce the production of GalNAc-DSLc4, suggesting that this enzyme is responsible for the synthesis of GalNAc-DSLc4 from DSLc4 in the cultured cells.

### Establishment of GalNAc-DSLc4-expressing clones from a renal cancer cell line with transfection of B4GalNAc-T2 cDNA

After the transfection of VMRC-RCW cells with the expression vector pcDNA3.1/B4GalNAc-T2, two clones were established. Two clones transfected with pcDNA3.1 alone were also isolated. These B4GalNAc-T2-expressing clones showed RM2 antigen (Fig. 1E). Fifty μM D-*threo*-1-phenyl-2-decanylamino-3-morpholio-1-propanol (D-PDMP) treatments for cultured transfectants resulted in the disappearance of positive population, indicating that positive RM2 reactivity in flow cytometric assay was almost toward GalNAc-DSLc4: RM2 epitope-carrying glycolipid (Fig. 1F). We also investigated the thin layer chromatography (TLC) pattern of glycolipids from the transfectants and control cells (Supplemental Figure S1). A band of GalNAc-DSLc4 was recognized between the bands of GD1a and GD1b. A band of GD3 was also recognized.

### Effects of GalNAc-DSLc4 expression on cell proliferation, invasion and adhesion

**Figure 2:**
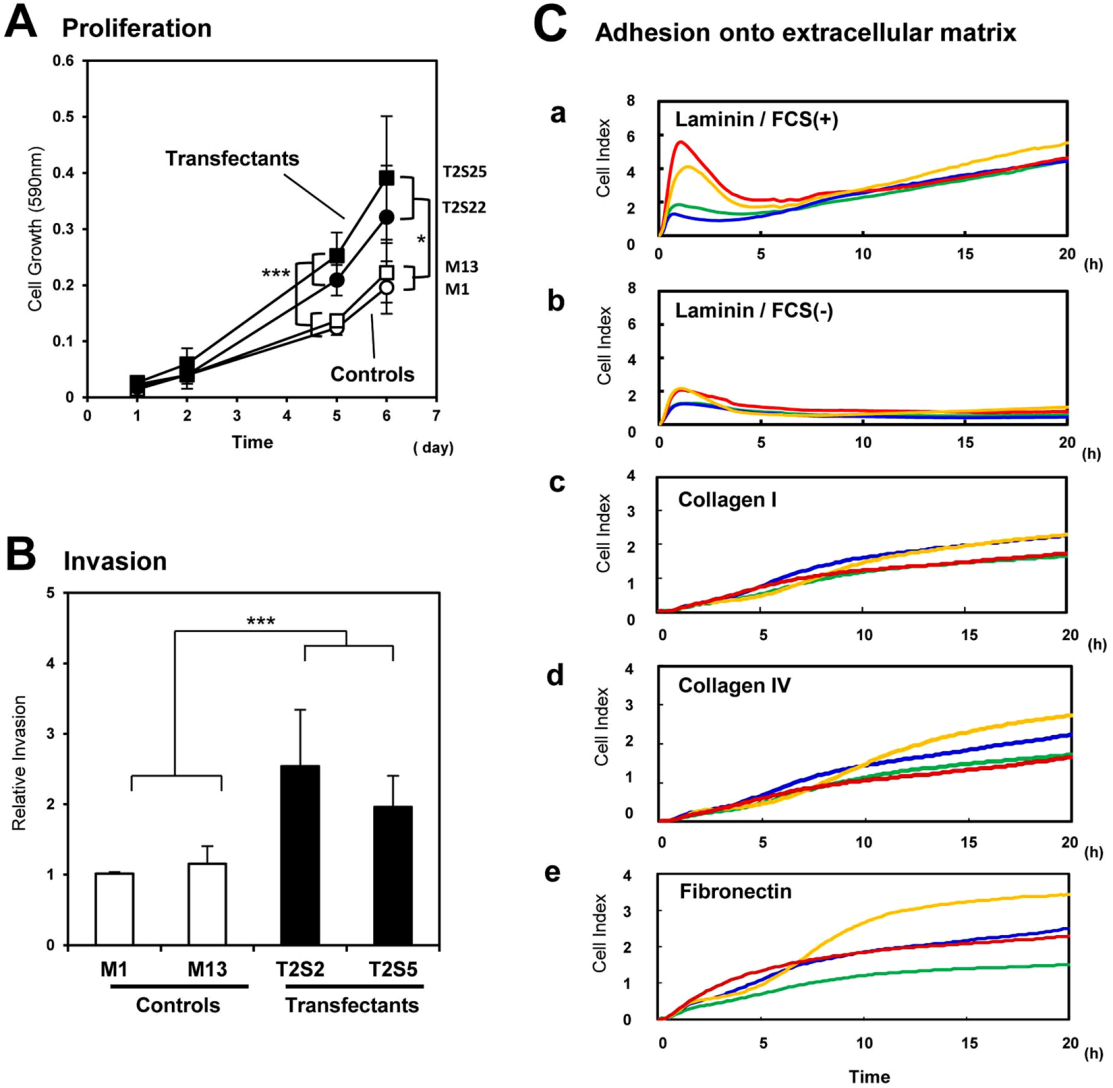
Malignant phenotypes of the B4GalNAc-T2 gene transfectant cells. (**A**) Effects of GalNAc-DSLc4 expression on the cell proliferation. Two transfectants and two vector controls (2.5 × 10^3^ cells/well) were seeded in 48-well plates in serum-containing medium and cultured for 6 days. The absorbance (590 nm) was measured on day 1, 2, 5, and 6. Data are means of three independent experiments. (**B**) Invasion activity of two transfectants and two vector controls. Cell invasion was analyzed by Boyden chamber invasion assay by counting the cell number on the reverse side of the filter. Data are means of three independent experiments. *Bars* indicate mean ± S.D. (n = 3). *P < 0.05, **P < 0.01, ***P < 0.005. (**C**) Dynamic monitoring of cell adhesion to LN, CL type I, CL type IV, or FN-coated surfaces. GalNAc-DSLc4-expressing cells and control cells were seeded in the wells of 96-well e-plate at 2.5 × 10^4^ cells/well with FCS, and cell attachment and spreading were monitored by RT-CES system. The e-plates were pre-coated with LN (*a*), CL type I (*b*), CL type IV (*c*), or FN (*d*). *Red* and *yellow* lines mean transfectant cells, and *green* and *blue* lines mean control cells.

Using these clones, effects of GalNAc-DSLc4 expression on cell proliferation, invasion and adhesion were examined. The proliferation of the transfectant cells and the control cells was compared by MTT assay. The transfectant cells showed significantly increased cell growth as shown in Fig. 2A. Invasion activity as analyzed by Boyden chamber method was also markedly increased in the transfectant cells (Fig. 2B). Intensity of adhesion and spreading of transfectant cells and control cells was analyzed by real time cell electronic sensing (RT-CES) system (Fig. 2C). ACEA e-plates were coated with laminin (LN), collagen (CL) type I, CL type IV, or fibronectin (FN). Transfectant cells adhered to LN more strongly than control cells in the presence of fetal calf serum (FCS) (Fig. 2C(a)). Adhesion activity of both transfectant cells and control cells for LN were lower under FCS-free conditions than in the presence of FCS (Fig. 2C(b)). For CL type I, CL type VI or FN, the adhesion intensity was very low in either transfectant cells or control cells, and no significant difference was found between them (Fig. 2C(c–e).

### Increased phosphorylation of Akt in the transfectant cells during FCS treatment

**Figure 3:**
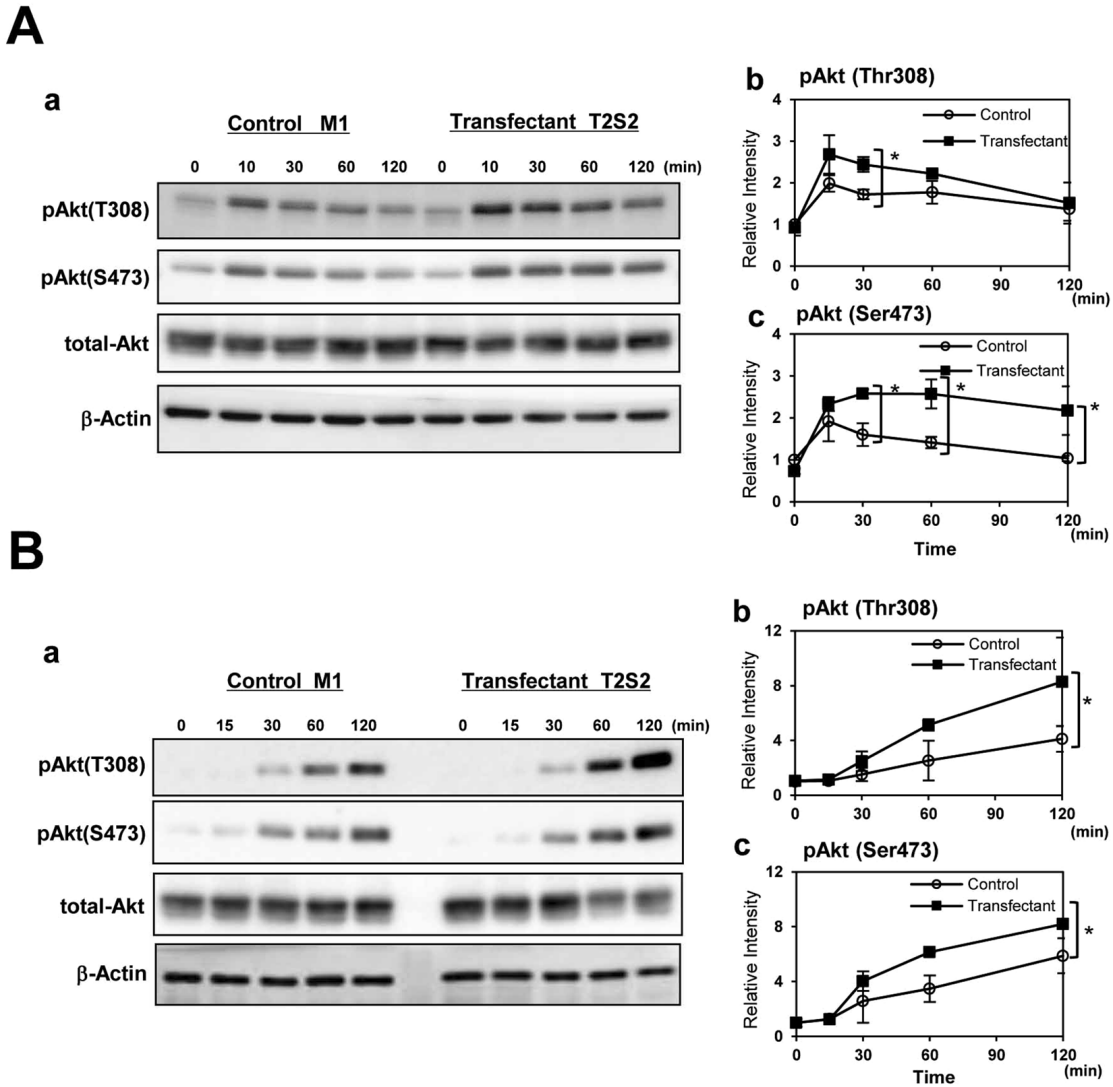
Integrin-ILK-Akt signaling was enhanced in GalNAc-DSLc4-expressing cells. (**A**) Phosphorylation of Akt during treatment with FCS in control cells and GalNAc-DSLc4 expressing cells was examined. Cells were prepared as described in “Materials and Methods”, and cell suspension (4 × 10^5^ cells) were added to plates, and incubated for 0, 10, 30, 60, or 120 min. After incubation, cells were lysed and used for immunoblotting using anti-phospho-Akt (Thr308), anti-phospho-Akt (Ser473), or anti-total Akt antibodies. Bands in autofluorograms (*a*) were quantified by a scanner, and the relative intensities of the bands were plotted after correction with total Akt bands (*b* and *c*). (**B**) Phosphorylation of Akt during adhesion to LN in GalNAc-DSLc4-expressing cells was examined. Cells (4 × 10^5^) were added to pre-coated plates with LN, and incubated for 0, 15, 30, 60, or 120 min. After incubation, cells were lysed and used for immunoblotting using anti-phospho-Akt (Thr308), anti-phospho-Akt (Ser473), or anti-total Akt antibodies. Bands in autofluorograms (*a*) were quantified by a scanner, and the relative intensities of the bands were plotted after correction with total Akt bands (*b* and *c*). *Bars* indicate mean ± S.D. (n = 3). *P < 0.05, **P < 0.01, ***P < 0.005. All cropped blots were run under the same experimental condition. The full-length blots are included in Supplemental Figure S7 respectively.

To analyze critical proteins involved in the increased proliferation and invasion of the transfectant cells, immunoblotting was performed with antibodies towards various anti-phosphorylated proteins. After cells were treated with FCS, cells were lysed and the lysates were immunoblotted using anti-phosphorylated Akt (Thr308 or Ser473) antibodies. Strongest levels of phosphorylation bands of Akt as detected by anti-phosphorylated Akt (Thr308) antibody appeared at 10 min incubation (Fig. 3A(a)). When cells were treated with various recombinant growth factors, levels of two phosphorylated forms of Akt were higher in the transfectant cells than in control cells, although the intensities of tyrosine phosphorylation of the growth factor receptors were weaker in the transfectant cells than in control cells (Supplemental Figure S2A and B).

### Increased phosphorylation of Akt in the transfectant cells during adhesion to LN

To investigate integrin signaling triggered by cell adhesion to LN, we analyzed phosphorylation of Akt in the transfectant and control cells. After serum starvation and rotation using a tube rotator, cells were plated in dishes pre-coated with LN under FCS (+) condition, and incubated at 37 °C for 0, 15, 30, 60, and 120 min (Fig. 3B). After incubation, cells were lysed and the lysates were immunoblotted using anti-pAkt antibodies. Notably, in the case of cell adhesion to LN, pAkt (Ser473) was activated from 30 min and pAkt (Thr308) was more strongly activated at 120 min in the transfectant cells.

### Integrin-ILK-Akt signaling pathway was enhanced in the transfectant cells

**Figure 4:**
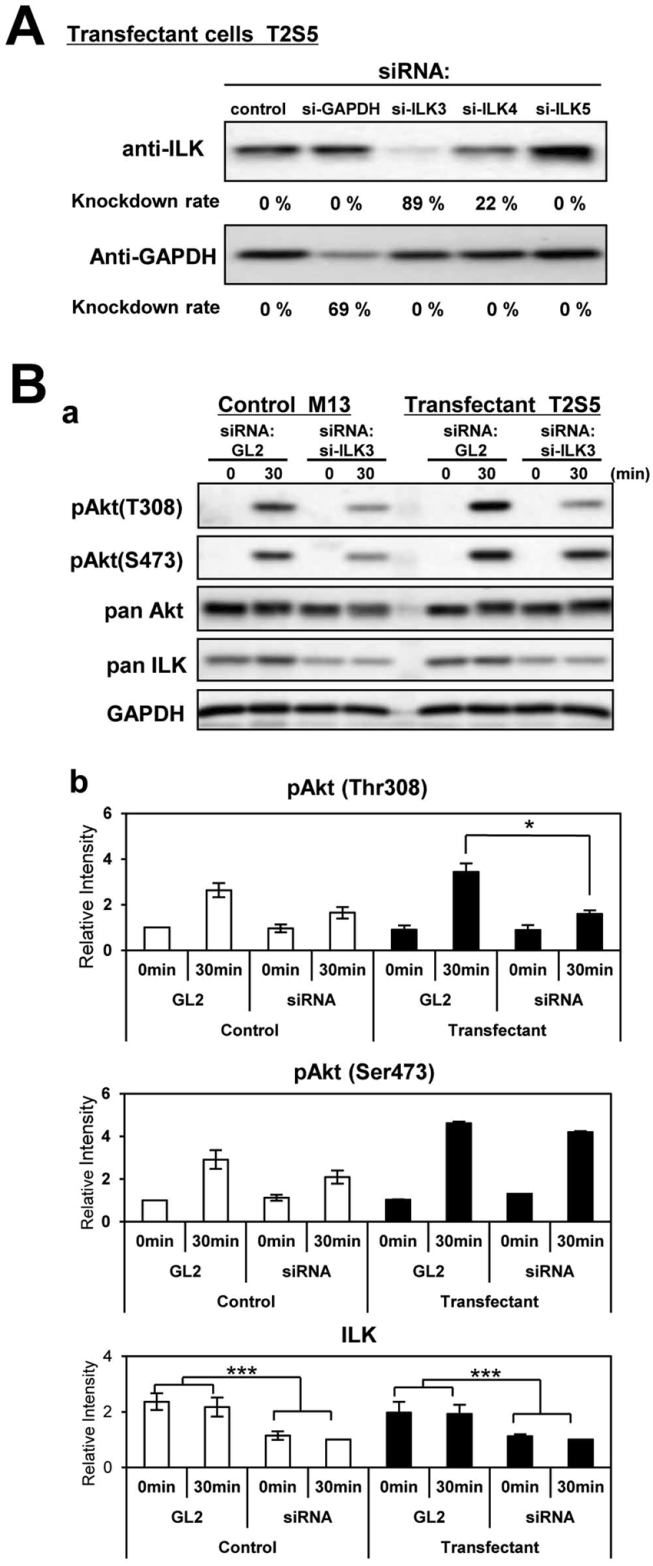
Reduction of Akt phosphorylation by ILK silencing. (**A**) Effects of siRNA for the knockdown of ILK on phosphorylation levels of Akt. Three kinds of anti-ILK siRNA and anti-GAPDH si-RNA as a control were analyzed. Si-ILK3 suppressed ILK levels efficiently (80~90%), and GL2 (anti-firefly luciferase siRNA) was used as a control. (**B**) Effects of ILK knockdown on phosphorylation levels of Akt in control cells and transfectants cells during adhesion to LN were examined. After 48 h of transfection, cells were plated on pre-coated LN, and lysed for immunoblotting at incubation time 0 and 30 min. Definite reduction in Akt phosphorylation was detected in the transfectant cells. *Bars* indicate mean ± S.D. (n = 3). *P < 0.05, **P < 0.01, ***P < 0.005. These were representative results among experiments repeated at least 3 times with similar results (**A**,**B**). All cropped blots were run under the same experimental condition. The full-length blots are presented in Supplemental Figure S7.

To examine the involvement of integrin-linked kinase (ILK) in Akt activation, siRNA: si-ILK3 was used for its highest efficiency (80~90%) in knockdown of ILK (Fig. 4A). The ILK-Akt signaling pathway was enhanced during cell adhesion to LN in the transfectant cells. In the transfectant cells treated with si-ILK3, stronger suppression in band intensities of pAkt (Thr308) was observed at 30 min after cell adhesion to LN than in those of control cells (Fig. 4B(a and b)).

### Intracellular localization of integrin β1 and caveolin-1 during stimuli of FCS and spatio-temporal relationships between integrins and GalNAc-DSLc4 during adhesion

**Figure 5:**
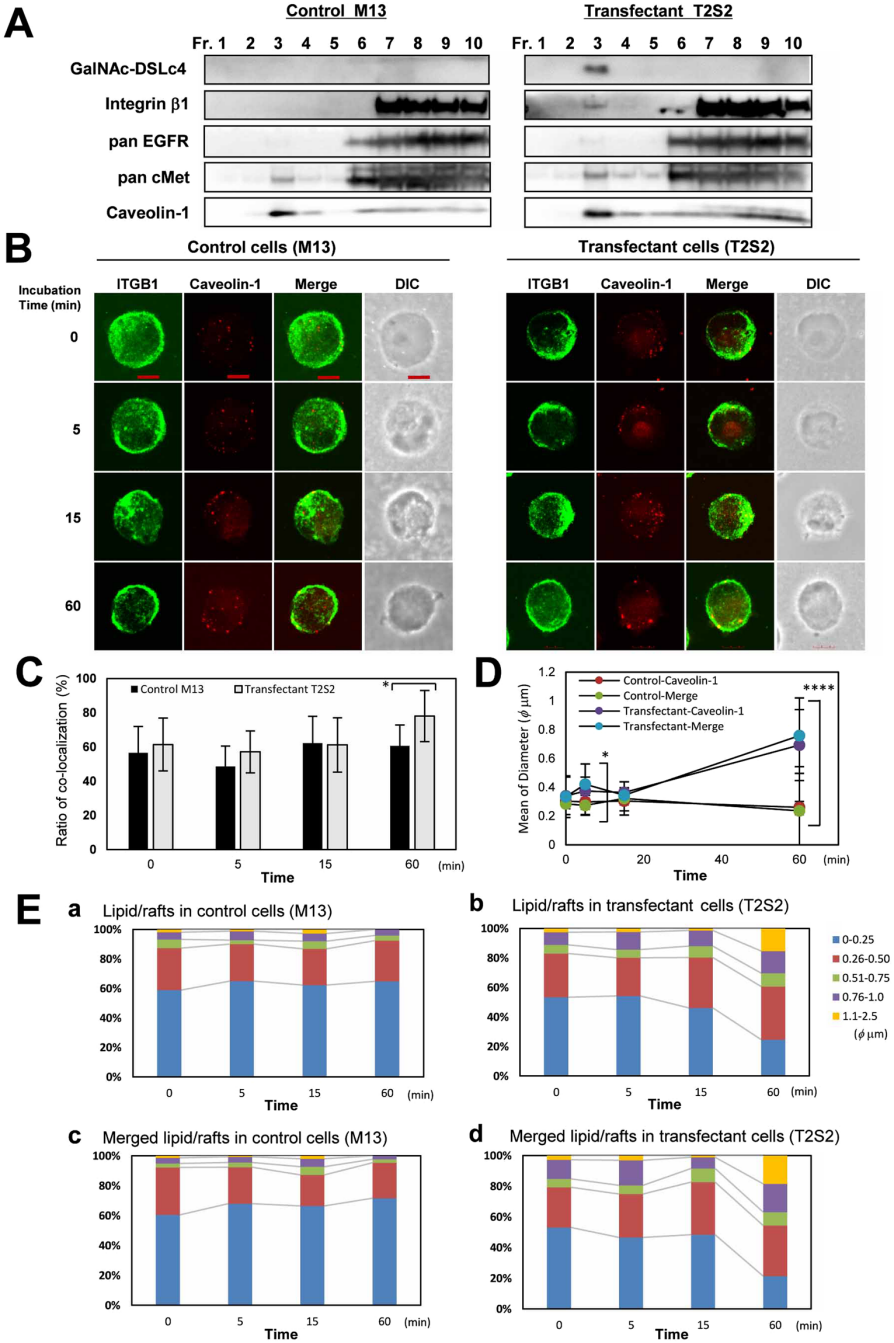
Intracellular localization of integrin β1 and caveolin-1 during FCS stimulation. (**A**) Integrin β1 was found in GEM/rafts during adhesion to LN-coated surface in the transfectants. Cells were lysed after incubation for 15 min at 37 °C, and separated by sucrose density gradient ultracentrifugation, and used for immunoblotting using anti-integrin β1, anti-EGFR, anti-cMet, or anti-caveolin-1 antibodies. Fractions 1–4 contained low density fractions and fractions 6–10 corresponded to high density fractions. These were representative results among experiments repeated at least 3 times with similar results. All cropped blots were run under the same experimental condition. The full-length blots are presented in Supplemental Figure S7. (**B**) The clones were immunocytostained with anti-integrin β1, anti-caveolin-1, and RM2 antibodies during FCS stimulation and adhesion onto LN. Cells were rotated under serum-free conditions for 30 min, and stimulated with 10% FCS. After incubation for 0, 5, 15, 60 min, cells were fixed in 4% paraformaldehyde for 10 min. Then, cells were stained for integrin β1 (ITGB1, *green*) and caveolin-1 (*red*), and their images were observed using a confocal microscope (Fluoview FV10i-DOC). *DIC*, image of differential interference contrast microscope. *Scale bar* indicate 10 μm. (**C**) Ratio of co-localization of integrin β1/caveolin-1. The number of dots (*red*) stained with anti-caveolin-1 antibody and the number of merged dots (*yellow*) stained with anti-integrin β1/caveolin-1 antibodies were counted, and percentage was obtained. The *column* represent mean ± S.D. (*n* = 10). *P < 0.01. (**D**) Mean of diameter of co-localized integrin β1/caveolin-1 dot areas. The diameters of merged dots (*yellow*) were measured, and the average was obtained. Values are means ± S.D. (*n* = 10). *P < 0.05, ****P < 0.0005. (**E**) Size distribution of lipid/rafts in control cells (*a*) and transfectant cells (*b*). Size distribution of merged dot area in control cells (*c*) and transfectant cells (*d*). The over 250 vesicular profiles were measured. *Blue*: < 0.025 μm, *red*: 0.26–0.50 μm, *green*: 0.51–0.75 μm, *purple*: 0.76–1.0 μm, *light blue*: 1.1–2.5 μm. In all experiments, it was confirmed that there was no cross-reaction between the individual antigens and non-relevant second reagents.

We examined the mechanism for enhanced ILK-Akt signaling in the transfectant cells by analyzing intracellular distribution of receptors, GalNAc-DSLc4 glycolipid, and integrin β1 using fractions from sucrose density ultracentrifugation (Fig. 5A). Caveolin-1 was used as a marker of GEM/rafts. Majority of GalNAc-DSLc4 glycolipid was detected in GEM/rafts. It is noteworthy that a part of integrin β1 was present in GEM/raft fraction in the transfectant cells. EGF receptor and cMet were also slightly increased in the GEM/rafts of the transfectant cells than in those of control cells. To clarify the spatio-temporal relationships between integrin β1 and GalNAc-DSLc4 in GEM/rafts, we examined changes of phases in the formation of GEM/rafts and the co-localization of integrin β1 and caveolin-1 with co-immunostaining during FCS stimuli, and the co-localization of integrin β1 and GalNAc-DSLc4 antigen with co-immunostaining during adhesion to LN.

Cells were treated and double-immunocytostained as described in Methods. Although the intracellular localization of integrin β1 seemed to show little change on cell surface due to their abundant expression, there were changes in the localization and formation of larger size of GEM/rafts on cell surface at 60 min after FCS stimuli in the transfectant cells (Fig. 5B). Ratio of co-localization of integrin β1/caveolin-1 and the diameter of GEM/rafts were increased at 60 min in transfectant cells (Fig. 5C and D). Moreover, we resorted to determine the diameter distribution for control cells and transfectant cells, and observed increased sizes of GEM/rafts in transfectant cells, especially at 60 min (Fig. 5E).

To investigate the co-localization of integrin β1 and GalNAc-DSLc4 antigen during adhesion to LN, cells were treated and double-immunocytostained as described in Methods. The localization of integrin β1 and GalNAc-DSLc4 antigen at various stages of adhesion is shown in Supplemental Figure S3A. Integrin β1 and GalNAc-DSLc4 antigen were uniformly stained at or around the adhesion sites at 5 min, and also stained at periphery of adhesion site at 30 min and 60 min with the progress of adhesion to LN (Supplemental Figure S3B). The adhesion mode onto pre-coated plate with LN was significantly different from that onto non-coated plate as shown at the bottom of Supplemental Figure S3B.

### Suppression of cell growth, invasiveness and adhesion activity in the transfectant cells by LY294002

**Figure 6:**
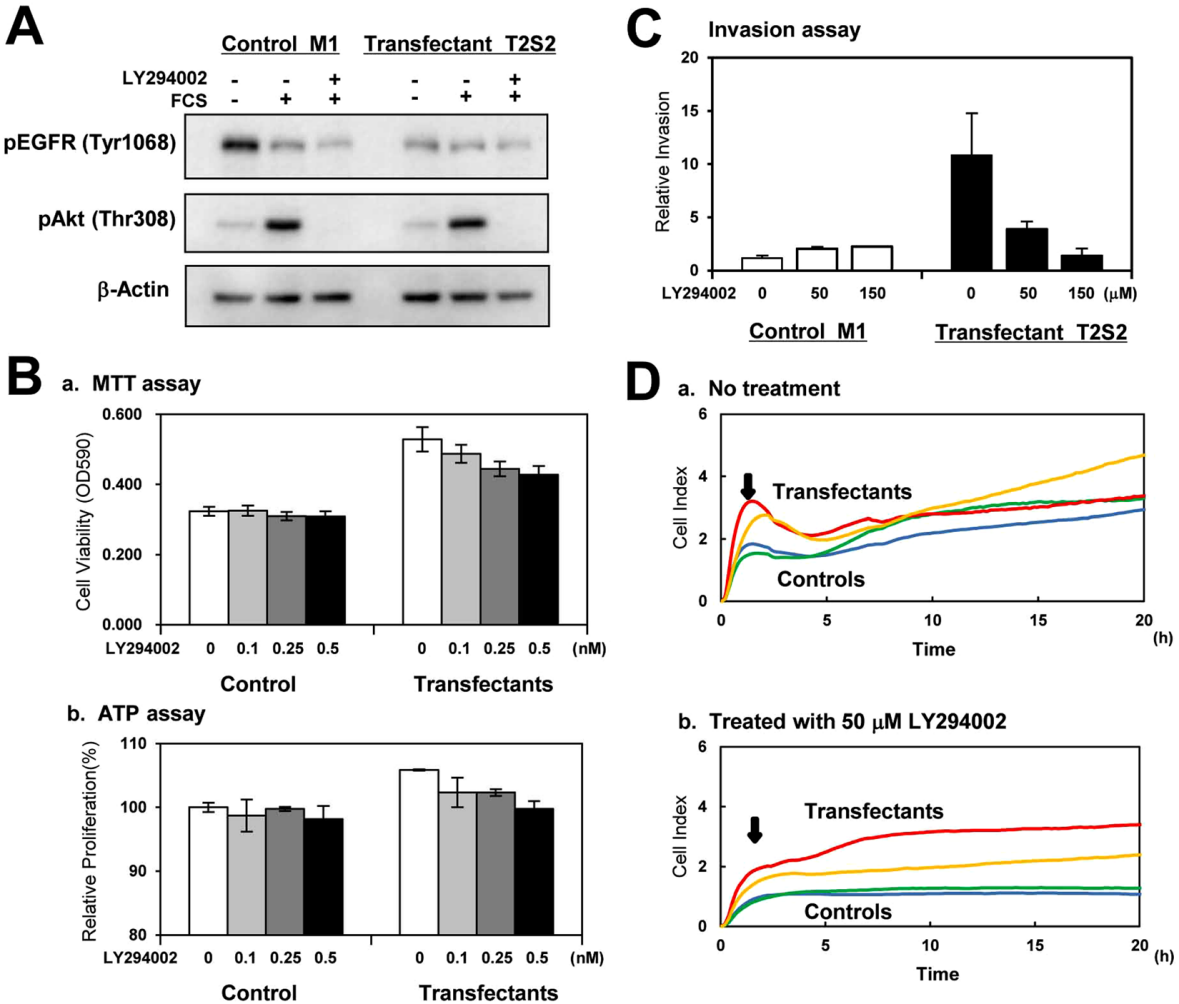
A PI3K inhibitor suppressed Akt phosphorylation and malignant properties of the transfectant cells. (**A**) Cells were cultured in serum free-medium overnight, and then treated with 50 μM LY294002 for 30 min. After treatment with serum-containing D-MEM for 5 min, cell lysates were prepared as described in “Materials and Methods”. Cropped blots were used here and uncropped imagesof blots are shown in Supplemental Figure S7 respectively. (**B**) To compare cell proliferation, MTT assay and ATP assay were performed. For MTT assay, transfectant cells and control cells (5.0 × 10^3^ cells/well) were prepared in 48-well plates in serum-containing D-MEM and cultured for 5 days (*a*). For ATP assay, transfectant cells and control cells (7.5 × 10^2^ cells/well) were prepared in 96-well plates in serum-containing D-MEM and cultured for 4 days (*b*). *Bars* indicate mean ± S.D. (n = 3). (**C**) For invasion assay, transfectant cells and control cells (2.5 × 10^4^ cells/well) were cultured in serum-containing D-MEM for 16 h. *Bars* indicate mean ± S.D. (n = 3). (**D**) For adhesion assay to laminin, the transfectant cells and control cells (1.0 × 10^4^ cells) were treated with 50 μM LY294002 for 22 h and seeded in the wells of e-plates. *Red* and *yellow* lines mean transfectant cells, and *green* and *blue* lines mean control cells.

Since it was suggested that the PI3K/Akt pathway was involved in the various enhanced phenotypes in the transfectant cells, an inhibitor of PI3K/Akt (LY294002) was used to treat the transfectant cells and control cells as described in “Materials and Methods”. Results showed that phosphorylation levels of both Akt (Thr308) and Akt (Ser473) were efficiently suppressed by treatment with 50 μM LY294002 (Fig. 6A). Then, the cell growth after addition of 0.1–0.5 nM LY294002 was examined. The proliferation of transfectant cells was suppressed dose-dependently by treatment with LY294002 in MTT assay (Fig. 6B(a)) and ATP assay (Fig. 6B(b)), but the proliferation of control cells was not. The invasiveness of the transfectant cells after treatment with 50 μM or 150 μM LY294002 was also suppressed dose-dependently as shown in Fig. 6C. We also analyzed the adhesion activity of the transfectant cells treated with 50 μM LY294002 for 22 h onto laminin by RT-CES system. The adhesion profile of the transfectant cells to LN became similar to the profile of the control cells (Fig. 6D). These results suggested that PI3K/Akt pathway is indeed involved in the enhanced proliferation and invasiveness of the transfectant cells.

### Suppression of cell growth, invasiveness, and adhesion activity in the transfectant cells by an anti-GalNAc-DSLc4 antibody

**Figure 7:**
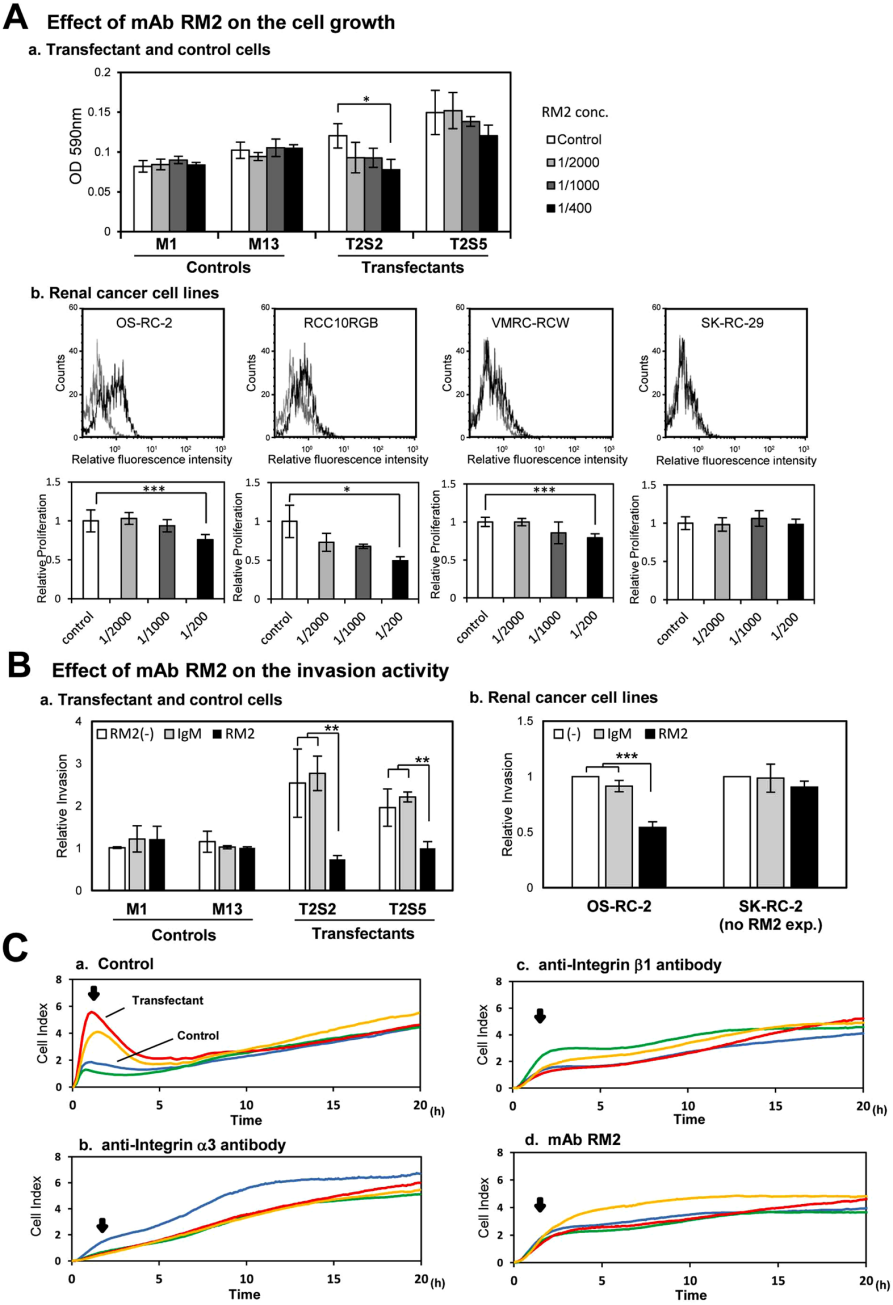
mAb RM2 suppressed malignant properties of the transfectant cells. (**A**) To investigate the effects of mAb RM2 on the cell growth of the transfectant cells and control cells, cells (2.5 × 10^3^ cells/well) were prepared in 48-well plates in serum-containing D-MEM and cultured for 5 days with mAb RM2-containing D-MEM. MTT assay was performed as described in “Materials and Methods” (*a*). The absorbance (590 nm) was measured on day 5. Data are means of three independent experiments. In a same manner, the effects of mAb RM2 on 4 RCC lines (OS-RC-2, RCC10RGB, VMRC-RCW, and SK-RC-29) were examined (*b*). Surface expression of GalNAc-DSLc4 on these cell lines was analyzed by flow cytometry using mAb RM2, and MTT assay with mAb RM2-containing D-MEM was performed as described above. *Black* profiles mean GalNAc-DSLc4 expressing cells, and *light gray* profiles mean negative control cells in flow cytometric assay. Data of MTT assay are means of three independent experiments. (**B**) The effect of mAb RM2 on invasiveness was investigated the transfectant cells or control cells using *in vitro* invasion assay (*a*). In same manner, effects of mAb RM2 on 2 RCC lines (OS-RC-2 and SK-RC-29) were examined (*b*). *Bars* indicate mean ± S.D. (n = 3). *P < 0.05, **P < 0.01, ***P < 0.005. (**C**) RT-CES of cells treated with antibodies. Suppression of adhesion to LN-coated plates by anti-integrin Abs or mAb RM2. Transfectant cells and control cells were seeded in the wells of 96-well e-plates at 2.5 × 10^4^ cells/well with FCS. RT-CES was performed as described in Fig. 2. *Red* and *yellow* lines mean transfectant cells, and *green* and *blue* lines mean control cells.

Effects of an anti-GalNAc-DSLc4 monoclonal antibody (mAb RM2) on cell growth were examined by adding the antibody to the culture medium. The increased cell growth after the transfection of B4GalNAc-T2 cDNA was suppressed in the presence of mAb RM2. The suppression effects were dependent on the concentration of the added antibody (Fig. 7A(a)). These suppression effects of mAb RM2 on the cell growth were also detected in other RCC lines (RCC10RGB, OS-RC-2, and VMRC-RCW), which were primarily expressing GalNAc-DSLc4, while it was not detected in a non-GalNAc-DSLc4-expressing line (SK-RC-29) as shown in Fig. 7A(b). In the same manner, when the invasion assay was performed after treatment with mAb RM2 for 30 min, we detected definite suppression of invasiveness by mAb RM2 both in the transfectant cells (Fig. 7B(a)) and in OS-RC-2 cells expressing GalNAc-DSLc4 (Fig. 7B(b)).

We also examined the inhibition effects on cell adhesion onto LN surface by anti-integrin α3 antibody, anti-integrin β1 antibody and mAb RM2. The anti-integrin α3 antibody suppressed strong adhesion of transfectant cells to LN as shown in Fig. 7C(b). We also detected the suppression of initial adhesion behavior by anti-integrin β1 antibody and by mAb RM2 (Fig. 7Cc and Cd) in the transfectant cells compared to controls (Fig. 7C(a)).

All results described above were summarized in Supplemental Figure S6. High expression of GalNAc-DSLc4 antigen might enhance integrin α3β1 functions, resulting in enhancement of cell proliferation, cell invasion and increased adhesion to laminin via PI3K/Akt pathway.

## Discussion

An initial study of glycosphingolipids in renal cell carcinoma (RCC) revealed that expression of GM2 was enhanced or a ganglioside migrating slower than GM2 on TLC plate was increased^16^. Fukushi *et al*. reported the expression of disialyl Lc4 in many tumor cells based on studies using a specific mAb^17^. Further, GalNAc-DSLc4, a derivative of disialyl Lc4, was isolated as a novel glycoshingolipid from TOS-1 cells which was derived from back metastatic lesion of an RCC patient^13,18^. Later, GalNAc-DSLc4, was shown to be a renal cancer-associated antigen and be recognized with RM2 antibody. Maruyama *et al*. reported that patients with RM2 antigen-positive cancers showed significantly poorer prognosis^19^. We also confirmed that about 70% of renal cancer cell lines (RCCs) express GalNAc-DSLc4 antigen using flow cytometric analysis with RM2 antibody (Table 1).

The mechanisms for the expression and roles of GalNAc-DSLc4 in RCCs have not been demonstrated to date. Therefore, our study started from experiments to identify B4GalNAc-T responsible for its biosynthesis. We examined the mechanisms for the synthesis of GalNAc-DSLc4 using already-cloned β4GalNAc transferase cDNAs. Among six cDNAs of B4GalNAc-T family, B4GalNAc-T1 is well-known as GM2/GD2 synthase, and B4GalNAc-T2 is responsible for the synthesis of Sd^a^ and Cad antigens. B4GalNAc-T3 and -T4 were cloned as synthases for LacdiNAc. Chondroitin sulfate (CS)-B4GalNAc-T1 and CS-B4GalNAc-T2 exert for the synthesis of chondroitin sulfate. In particular, transferase activity of B4GalNAc-T2 has been described mainly in human intestine^20^, colon^21^, and kidney^22^. Thus, GalNAc-T2 is expressed in normal GI tracts, while it is expressed in a cancer-specific manner in RCC. Kawamura Y. I. *et al*.^23^ found that introduction of the *B4GalNAc-T2* gene essentially inhibited the expression of SLe^x^ and SLe^a^ from both glycoprotein and glycolipid components in gastrointestinal cancer cells. In the present study, it was demonstrated that B4GalNAc-T2 was responsible for the synthesis of GalNAc-DSLc4 from DSLc4 in RCC as we expected (Fig. 1A). The possibility that GalNAc-sialyl Lc4 can be a precursor of GalNAc-DSLc4 is very low as shown in Supplemental Figure S4. The B4GalNAc-T2 gene expression levels well correlated with the expression levels of GalNAc-DSLc4 in RCCs examined (Supplemental Figure S5). On the other hands, this enzyme was scarcely detected in colon cancer cell lines as it has been noted that the expression of GalNAc-T2 mRNA transcript is drastically reduced in oncogenetic processes in gastrointestinal tissue^23^.

The stable transfectants of GalNAc-T2 showing high expression of GalNAc-DSLc4, exhibited increased proliferation and invasion activities, and specific adhesion to laminin-coating plates (Fig. 2). The malignancy of transfectants was enhanced based on the activation of PI3K/Akt signaling pathway by serum stimulation or adhesion to laminin as shown in Fig. 3. It was reasonable that the treatment of transfectant cells with LY294002, an inhibitor for PI3K, could suppress the phosphorylation of Akt and also increased malignant features of the transfectant cells (Fig. 6).

These results suggested that GalNAc-DSLc4 mediate malignant signals triggered by serum or adhesion to laminin by modulating receptor functions for individual stimulants on the cell surface. Actually, mAb RM2 reactive with GalNAc-DSLc4 inhibited increased malignant properties such as high proliferation, increased invasiveness, and strong adhesion of GalNAc-DSLc4-expressing cells (Fig. 7), suggesting that GalNAc-DSLc4 is involved in the enhancement of malignant properties of RCCs. Co-localization of GalNAc-DSLc4 and integrins in GEM/Rafts suggested physical assoc**i**ation of them on the cell surface of RCC cells, leading to generation of novel signaling (Fig. 5).

K. Simons *et al*. reviewed advances in technology that pointed to the existence of raft-based membrane heterogeneity in living cells and discussed the levels of preferential association underlying dynamic domain structure and biological function(s)^24,25^. They argued that lipid rafts are fluctuating nanoscale assemblies of sphingolipid, cholesterol, and proteins that can be stabilized to coalesce, forming platforms that function in membrane signaling and trafficking. Expression of GalNAc-DSLc4 in transfectant cells are interpreted as being equipped with a glycosphingolipid-dependent bias in composition at the nanoscale, allowing partitioning to and assembly of more stable raft platforms in the functionalized state. Furthermore, because raft activation is often stabilized or nucleated by scaffolding elements such as cortical actin^26^, integrin β1 and GalNAc-DSLc4 antigen were dramatically co-stained at or around the adhesion sites and at periphery of adhesion site with the progress of adhesion to LN as shown in Supplemental Figure S3. The interaction of integrin family and extracellular matrix is often considered as an important event for attachment of tumor cells onto distant organs, while the alteration of the cadherin family, which is important in the cell-cell adhesion, is essential for detachment from primary tumor sites.

There are some reports indicating that integrin β1 is important in tumor cell migration and recognition of extracellular matrix, and that gangliosides might be involved in the increased/decreased proliferation and invasion via enhancement/suppression of integrin signaling. GD3 expression in melanoma cells enhanced malignant properties, such as cell growth or invasion activity, based on increased integrin functions^27–30^. Generally, disialogangliosides enhance malignant properties although monosialyl compounds work inversely^31^. Indeed, gangliosides GM3 or GM2 were associated with reversion from oncogenic to normal cell phenotypes by forming complex with tetraspanin CD9 or CD82 in microdomains. The formation of GM3/CD9/integrin α3 complex in bladder cancer cells suppressed malignant phenotypes^32^. GM2 formed a complex with CD82, and interacted with Met and thereby inhibited HGF-induced Met tyrosine kinase activity, as well as cross-talk of integrin with Met^33^. As for highly sialylated gangliosides, they appeared to regulate integrin α5β1-mediated adhesion of epithelial cells to fibronectin through carbohydrate-carbohydrate interactions^34^.

In this study, we found that crosstalk between integrins and growth factor receptors was induced by the presence of GalNAc-DSLc4, a branched-type disialyl antigen in RCCs. Actually, stronger phosphorylation of Akt during adhesion could be observed in GalNAc-DSLc4-expressing cells (Fig. 3), and knockdown of ILK resulted in the reduction of Akt phosphorylation levels (Fig. 4). This fact suggested that ILK plays a role as an adaptor molecule, causing Akt activation.

We detected suppression of cell growth and invasiveness by mAb RM2, and also effective inhibition of initial adhesion to LN by mAb RM2 as well as anti-integrin α3 antibody and anti-integrin β1 antibody (Fig. 7C). These branched disialyl structures such as disialyl Le^a^, disialyl Lc4, DSGG and GalNAc-DSLc4 might play important roles for the complex formation of integrins/growth factor receptos/glycosphingolipids in microdomains.

The trial to validate high metastatic potential of GalNAc-DSLc4-expressing RCC is now on going in our laboratories. The metastatic cells among RCCs might be associated with overexpression of GalNAc-DSLc4, being involved in disialyl antigen-dependent adhesion of tumor cells to lung, leading to the activation of signals to enhance invasiveness^35^.

Many types of disialyl gangliosides and sialylated *O*- or *N*-linked glycans are targets of variety of siglecs (sialic acid-binding immunoglobulin-like lectins) with different specificities^36,37^. Ito *et al*. has studied the binding specificity of Siglec-7 to DSGb5^38^. To clarify the functional role of DSGb5 in RCC metastases, Kawasaki *et al*. investigated whether DSGb5 expressed on RCC cells can modulate NK cell cytotoxicity in a Siglec-7-dependent manner. It was found that DSGb5 expressed on RCC cells can downregulate NK cell cytotoxicity in a DSGb5-Siglec-7-dependent manner, suggesting that RCC cells with DSGb5 create favorable environments for their own survival and metastases^39^. The results demonstrated in this study suggest that GalNAc-DSLc4 might be also play roles as a ligand recognized by Siglecs, and be involved in the generation of inhibitory signals in immune cells.

The disappearance of positive RM2 reactivity with D-PDMP treatments of cultured transfectants in flow cytometric assay suggested that RM2-related epitopes scarcely exist on *O*-glycans or *N*-glycans (Fig. 1F). However, it is still possible that B4GalNAc-T2 transfers a GalNAc onto glycan epitope in glycoproteins expressed in RCCs. Changes in the glycan-epitopes on proteins are under investigation.

On the other hand, the GalNAc-DSLc4 antigen can also be developed as a novel marker for prostate cancer, because the GalNAc-DSLc4 antigen is highly expressed on the cancer cell surface as a glycolipid and possibly as a glycoprotein^40^. Chuang *et al*. have succeeded the chemical synthesis of the GalNAc-DSLc4 antigen and also reported the study of vaccine evaluation with GalNAc-DSLc4 antigen as a prostate cancer-associated carbohydrate antigen^41^. In our work on RCCs, we, for the first time, elucidated substantial basis for the biosynthetic reaction occurring *in vivo*, and reported the biological function of GalNAc-DSLc4 antigen using cells established by genetic manipulation. Consequently, the GalNAc-DSLc4 antigen on the cell surface in RCCs might be a very specific and promising tumor marker, and also a candidate for molecular targets of cancer therapeutics to eradicate highly metastatic cancer cells. Further understanding of both of the structural basis and biological significance of GalNAc-DSLc4 antigen will contribute to the development of therapeutic methods.

## Methods

Details are described in Supplementary Methods.

### Cell lines

Human renal cancer cell lines (SK-RC 1, SK-RC 6, SK-RC 7, SK-RC 17, SK-RC 29, SK-RC 35, SK-RC 39, SK-RC 44, SK-RC 45, SK-RC 99 and Moroff) were established at Memorial Sloan-Kettering Cancer Center (New York, NY, U.S.A). Human renal cancer cell lines (Caki-1, VMRC-RCW, VMRC-RCZ, TUHR10TKB and TUHR14TKB) were obtained from the Human Science Research Resources Bank (Osaka, Japan). Human renal cancer cell lines (OS-RC-2, RCC10RGB, TUHR4TKB) were obtained from the Riken Cell Bank (Wako, Japan). A human renal cancer cell line, ACHN, was purchased from Dainihonseiyaku Co. Normal HRPTE were purchased from Sanko Junyaku Co. All human renal cancer cell lines were grown in Dulbecco’s modified Eagle’s medium (D-MEM) supplemented with 10% (v/v) FCS. HRPTE cells were cultured in renal epithelial cell growth medium Bullet Kit^TM^ (Sanko Junyaku Co.). Mouse fibroblast L cells was grown in D-MEM supplemented with 7.5% FCS.

### Antibodies

Monoclonal antibodies (mAbs) used were as follows: anti-MSGG (RM1), anti-DSGG (5F3) and anti-GalNAc-DSLc4 (RM2) were established as described previously^12^; anti-DSLc4 mAb (FH9) was kindly provided by R. Kannagi at Aichi Medical University, Nagakute. FITC-labeled anti-mouse IgG antibody and anti-mouse IgM antibody were purchased from ICN/Cappel (Durham, NC). Anti-rabbit IgG antibody conjugated with horseradish peroxidase (HRP) was purchased from Cell Signaling Technology (Beverly, MA). Anti-mouse IgG (H + L) conjugated with HRP was from BETHYL Laboratories, Inc. (Montgomery, TX). Anti-mouse IgG conjugated with HRP and anti-goat IgG conjugated with HRP were from Santa cruz biotechnology, Inc. (Dallas, TX). Anti-phospho-Akt (Thr308), anti-phospho-Akt (Ser473), anti-Akt, anti-phospho-EGFR (Tyr1068), anti-EGFR, anti-phospho-cMet (Tyr1234/1235), anti-phospho-cMet (Tyr1349), anti-cMet, anti-phospho-ERK p44/42 (Thr202/Tyr204), anti-ERK p44/42, anti-Caveolin-1, and anti-ILK, were purchased from Cell Signaling Technology (Beverly, MA). Goat polyclonal anti-integrin β1 antibody and mouse monoclonal anti-integrin α3 were purchased from Santa cruz biotechnology, Inc. Mouse anti-CD29 mAb was purchased from BD Transduction Laboratories (San Jose, CA). Monoclonal mouse anti-human CD29 (integrin β1)/biotin antibody was from Ancell (Bayport, MN). Anti-β-actin mAb conjugated with horseradish peroxidase and anti-GAPDH mAb conjugated with horseradish peroxidase were purchased from Wako Pure Chemical Industries, Ltd (Osaka, Japan).

### Construction of expression vectors

Plasmid DNA construction is described in Supplementary Methods.

### Flow cytometric analysis

Cell surface expression of glycolipids on 20 renal cancer cell lines and HRPTE cells was analyzed using mAbs by a FACScalibur^TM^ with CellQuest^TM^ version 3.1 f software (Becton Dickinson). Cells were incubated with mAbs for 1 h on ice and stained with FITC-conjugated second antibodies. Control cells for flow cytometry were prepared using the second antibody alone.

### Preparation of membrane fraction

L cells (3 × 10^6^) were plated in 10-cm dishes and transiently transfected with an expression plasmid (4 μg) by Lipofectamine^TM^ 2000 (Thermo Fisher Scientifi*c*) according to the manufacturer’s instructions. The cells were harvested after 48 h, and the membrane fraction was prepared using a nitrogen cavitation apparatus (Parr Instrument Co., Moline, IL) as described^11^.

### N-acetylgalactosaminyltransferase assay

The *N*-acetylgalactosaminyltransferase assay was performed as previously described^11^.

### Transfection for flow cytometric analysis

The THUR14TKB cells were transiently transfected by Lipofectamine^TM^ 2000 as described in Supplementary Methods.

### D-PDMP treatment of transfectant cells

D-PDMP (an inhibitor of glucosylceramide synthase^42^) was purchased from Sigma, and dissolved in distilled water at concentration of 4 mM. This stock solution was directly added to culture medium. The transfectant cells were treated with 50 μM D-PDMP for 6 days.

### Glycolipid extraction and TLC

Glycolipids were extracted as described previously^43^.

### MTT assay

For cell proliferation assay, transfectant cells and control cells (2.5 × 10^3^ cells/well) were prepared in 48-well plates in serum-containing medium and cultured for 6 days. MTT assay was performed by assessing the reduction of MTT to formazan based on the absorbance at 590 nm using Molecular Devices Vmax kinetic microplate reader^TM^ with SOFT max Pro.3.1.1^TM^ software (Molecular Devices). For cell growth inhibition assay, transfectant cells and control cells (2.5 × 10^3^ cells/well) were seeded in 48-well plates in serum-containing medium and treated with mAb RM2 antibody diluted to the indicated concentrations for 5 days.

### Cell viability assay

To determine cell viability, we quantify the amount of ATP present, which indicates the presence of metabolically active cells. Cells grown in 96-well plates for 4 days (7.5 × 10^2^ cells/well) were prepared. Then CellTiter-Glo™ Reagent (Promega Corporation, Madison, MI) was added with equal volume to that of cell medium in each well. Plates were incubated at room temperature for 10 min on a shaker and luminescence was measured on a luminometer (TriStar^2^ LB942, Berthold Technology) according to the manufacturer’s instruction.

### *In vitro* invasion assay

Invasion assay were performed using BioCoat™ Matrigel™ Invasion Chamber (Corning) as described previously^16^ with some modifications.

### Cell adhesion assay using real time cell electronic sensing (RT-CES)

ACEA e-plates (ACEA Biosciences, San Diego, CA) were coated with FN from human plasma, LN from human placenta, CL type I, or CL type IV from human placenta for 1 h at 37 °C. FN, CL type I, and CL type III were purchased from Chemicon (Temecula, CA). LN was purchased from Sigma. The plates were washed with PBS and coated with 0.5% BSA in PBS for 30 min at 37 °C. The wells were washed with PBS before the addition of culture medium with or without serum, and cells (2 × 10^4^) were added on ACEA e-plate coated with various extracellular matrix proteins. The adhesion of cells was continuously monitored using the RT-CES system (Wako Pure Chemical).

To examine the inhibition of cell adhesion by mAbs reactive with anti-integrin β1, anti-integrin α3, or mAb RM2, cells were pretreated with these mAbs for 30 min at room temperature before plating into wells.

### Integrin-mediated adhesion to laminin

Cells were starved for 14–16 h in serum-free D-MEM, and harvested with 0.02% EDTA in PBS. To reduce basal phosphorylation of signaling molecules, cells were rotated for 30 min at 37 °C using a tube rotator (TR-118, AS ONE, Osaka, Japan). Cell suspensions (4 × 10^5^) were added to 6-cm dishes pre-coated with LN from human placenta (Sigma-Aldrich, Germany). Cells were lysed after incubation at 37 °C, and lysates were used for Western immunoblotting.

### Western immunoblotting

Cells were lysed with cell lysis buffer (20 mM Tris-HCl, 150 mM NaCl, 1 mM Na_2_EDTA, 1 mM EGTA, 1% Triton X-100, 2.5 mM sodium pyrophosphate, 1 mM β-glycerophosphate, 1 mM Na_3_VO_4_, 1 μg/ml of leupeptin) (Cell Signaling), Protease Inhibitor Mixture^TM^ (Calbiochem, SanDiego, CA), and 1 mM PMSF. Insoluble materials were removed by centrifugation at 15,000 × g for 10 min at 4 °C. Cell lysates were separated by SDS-PAGE using 7.5% or 10% gels. The separated proteins were transferred onto a Fluoro Trans™ W Membrane (PALL, Dreieich, Germany). Blots were blocked with 3% BSA in PBS. The membrane was first probed with primary antibodies. After being washed with PBS containing 0.05% Tween 20, the blots were incubated with respective secondary antibodies conjugated with horseradish peroxidase. After the membrane was washed, bound conjugates were visualized with an enhanced chemiluminescence (ECL^TM^) detection system (PerkinElmer Life Sciences) and imaged using a Biorad ChemiDoc XRS™ (BioRad).

### Knockdown of ILK

Suppression of ILK with 3 kinds of siRNA was examined by immunoblotting using an anti-ILK antibody (Cell signaling) after 72 h of transfection into GalNAc-DSLc4-expressing cells as previously described^28^. GL2, siRNA for a firefly luciferase (B-Bridge International, Inc. CA) was used as a negative control. Si-GAPDH was also used as a control. si-ILK3 (5′AAGUUAAGCUGUUUGAAGUCAAUGC-3′) (Invitrogen) was the most effective (knockdown rate was about 80–90%).

### Preparation of GEM/raft fractions

GEM/rafts were isolated using a detergent extraction method as described previously^17,44^.

### Immunofluorescence staining

Cells were rotated under serum-free conditions for 30 min, and stimulated with 10% FCS followed by incubation for 0–60 min. Cells were fixed in 4% paraformaldehyde for 10 min at room temperature. After being washed with PBS, nonspecific binding was blocked with 2.5% normal donkey serum and 5% normal goat serum in PBS for 30 min at room temperature. Cells were incubated with biotin-conjugated anti-human CD29 antibody and anti-caveolin-1 antibody in PBS for 45 min at room temperature, then with streptavidin-Alexa 488 and anti-rabbit IgG-Alexa 594 in PBS for 30 min at room temperature. The resulting staining patterns were imaged using a confocal microscope (Fluoview FV10i-DOC, Olympus, Tokyo, Japan).

### PI3K inhibitor treatment

Cells were cultured with serum-free D-MEM overnight, and then treated with 50 μM or 150 μM LY294002 for 30 min. After treatement with serum-containing D-MEM for 5 min, the cell lysates were prepared and used for Western immunoblotting with anti-EGFR (Tyr1068) or anti-phospho Akt (Thr308) antibodies. In the same manner, 0–0.5 nM of LY294002-treated cells were harvested with 0.02% EDTA in PBS, and used for cell proliferation assay, 0–150 μM of LY294002-treated cells were used for invasiveness assay and 50 μM of LY294002-treated cells were used for adhesion assay.

### mAb RM2 treatment

Cells were cultured with serum-free D-MEM overnight, and mAb RM2-treated cells were harvested with 0.02% EDTA in PBS. The cells were treated with 1/200 conc. of stock solution (1 mg/ml) of mAb RM2 in serum-free D-MEM for 30 min, and were used for cell proliferation assay, invasiveness assay and adhesion assay.

### Statistical analysis

The statistical significance was examined by the Student’s *t*-test.

## Electronic supplementary material

Suplemental Information

## References

[1] Hakomori S (1996). Tumor malignancy defined by aberrant glycosylation and sphingo(glyco)lipid metabolism. Cancer Res..

[2] Hakomori S (2002). Glycosylation defining cancer malignancy: new wine in an old bottle. Proc. Natl. Acad. Sci. USA.

[3] Yoshida S (2001). Ganglioside GD2 in small cell lung cancer cell lines. Cancer Res..

[4] Thurin J (1986). GD2 ganglioside is a biochemical event in melanoma tumor progression. FEBS Lett..

[5] Kuo CT (1998). Assessment of messenger RNA of beta 1 → 4-N-acetylgalactosaminyl-transferase as a molecular marker for metastatic melanoma. Clin. Cancer Res..

[6] Nakano J, Raj BK, Asagami C, Lloyd KO (1996). Human melanoma cell lines deficient in GD3 ganglioside expression exhibit altered growth and tumorigenic characteristics. J. Invest. Dermatol..

[7] Angata T, Varki A (2000). Cloning, Characterization, and phylogenetic analysis of siglec-9, a new member of the CD33-related group of siglecs. Glycobiology..

[8] Nicoll G (1999). Identification and characterization of a novel siglec, siglec-7, expressed by human natural killer cells and monocytes. J. Biol. Chem..

[9] Okajima T (1999). Molecular cloning of brain-specific GD1α synthase (ST6GalNAc V) containing CAG/Glutamine repeats. J. Biol. Chem..

[10] Okajima T (2000). Molecular cloning and expression of mouse GD1α/GT1aα/GQ1bα synthase (ST6GalNAc VI) Gene. J. Biol. Chem..

[11] Tsuchida A (2003). Synthesis of disialyl Lewis a (Le^a^) structure in colon cancer cell lines by a sialyltransferase, ST6GalNAc VI, responsible for the synthesis of a-series gangliosides. J. Biol. Chem..

[12] Saito S, Levery SB, Salyan MEK, Goldberg RI, Hakomori S (1994). Common tetrasaccharide epitope NeuAc alpha 2 → 3Gal beta 1 → 3(Neu-Ac alpha 2→)GalNAc, presented by different carrier glycosylceramides or *O*-linked peptides, is recognized by different antibodies and ligands having distinct specificities. J. Biol. Chem..

[13] Ito A, Levery SB, Saito S, Satoh M, Hakomori S (2001). A novel ganglioside isolated from renal cell carcinoma. J. Biol. Chem..

[14] Montiel MD, Krzewinski-Recchi MA, Delannoy P, Harduin-Lepers A (2003). Molecular cloning, gene organization and expression of the human UDP-GalNAc: Neu5Acalpha2-3Galbeta-Rbeta1,4-N-acetylgalactosaminyltransferase responsible for the biosynthesis of the blood group Sda/Cad antigen: evidence for an unusual extended cytoplasmic domain. Biochem. J..

[15] Senda M (2007). Identification and expression of a sialyltransferase responsible for the synthesis of disialylgalactosylgloboside in normal and malignant kidney cells: downregulation of ST6GalNAc VI in renal cancers. Biochem. J..

[16] Karlsson KA, Samuelsson BE, Schersten T, Steen GO, Wahlqvist L (1974). The sphingolipid composition of human renal carcinoma. Biochim. Biophys. Acta..

[17] Fukushi Y, Nudelmanm E, Levery SB, Higuchi T, Hakomori S (1986). A novel disiloganglioside (IV3NeuAcIII6NeuAcLc4) of human adenocarcinoma and the monoclonal antibody (FH9) defining this disialosyl structure. Biochemistry.

[18] Satoh M (1999). Four new human renal cell carcinoma cell lines expressing globo-series gangliosides. Tohoku J. Exp. Med..

[19] Maruyama R (2007). High incidence of GalNAc disialosyl lactotetraosylceramide in metastatic renal cell carcinoma. Anticancer Res..

[20] Malagolini N (1989). Increased CMPNeuAc: Galβ1,4GlcNAc-R α2,6 sialyltransferase activity in human colorectal cancer tissues. Cancer Res..

[21] Morton JA, Pickles MM, Vanhegan RI (1988). The Sd^a^ antigen in the human kidney and colon. Immunol. Invest..

[22] Piller F, Blanchard D, Huet M, Cartron JP (1986). Identification of a alpha-NeuAc-(2-3)-beta-D-galactopyranosyl N-acetyl-beta-D-galactosaminyltransferase in human kidney. Carbohydr. Res..

[23] Kawamura YI (2005). Introduction of Sd(a) carbohydrate antigen in gastrointestinal cancer cells eliminates selectin ligands and inhibits metastasis. Cancer Res..

[24] Lingwood D, Simon K (2010). Lipid rafts as a membrane-organizing principle. Science.

[25] Simons K, Gerl MJ (2010). Revitalizing membrane rafts: new tools and insights. Nature Rev. Mol. Cell Biol..

[26] Viola A, Gupta N (2007). Tether and trap: regulation of membrane-raft dynamics by actin-binding proteins. Nat. Rev. Immunol..

[27] Hamamura K (2005). Ganglioside GD3 promotes cell growth and invasion through p130Cas and paxillin in malignant melanoma cells. Proc. Natl. Acad. Sci. USA.

[28] Ohkawa Y (2010). Ganglioside GD3 enhances adhesion signals and augments malignant properties of melanoma cells by recruiting integrins to glycolipid-enriched microdomains. J. Biol. Chem..

[29] Hamamura K (2008). Focal adhesion kinase as well as p130Cas and paxillin is crucially involved in the enhanced malignant properties under expression of ganglioside GD3 in melanoma cells. Biochim. Biophys. Acta.

[30] Ohkawa Y (2008). Essential roles of integrin-mediated signaling for the enhancement malignant properties of melanomas based on the expression of GD3. Biochem. Biophys. Res. Commun..

[31] Furukawa K, Hamamura K, Ohkawa Y, Ohmi Y, Furukawa K (2012). Disialyl gangliosides enhance tumor phenotypes with differential modalities. Glycoconj. J..

[32] Toledo MS, Suzuki E, Handa K, Hakomori S (2004). Cell growth regulation through GM3-enriched microdomain (glycosynapse) in human lung embryonal fibroblast WI38 and its oncogenic transformant VA13. J. Biol. Chem..

[33] Todeshini AR, Dos Snatos JN, Handa K, Hakomori S (2007). Ganglioside GM2-tetraspanin CD82 complex inhibits met and its cross-talk with integrins, providing a basis for control of cell motility through glycosynapse. J. Biol. Chem..

[34] Wong X (2001). Carbohydrate-carbohydrate binding of ganglioside to integrin α5 modulates α5β1 function. J. Biol. Chem..

[35] Satoh M (1996). Disialosyl galactosylgloboside as an adhesion molecule expressed on renal cell carcinoma and its relationship to metastatic potential. Cancer Res..

[36] Brinkman-Van der Linden EC, Varki A (2000). New aspects of siglec binding specificities, including the significance of fucosylation and of the sialyl-Tn epitope. Sialic acid-binding immunoglobulin superfamily lectins. J. Biol. Chem..

[37] Crocker PR, Varki A (2001). Siglecs in the immune system. Immunology.

[38] Ito A, Handa K, Withers DA, Satoh M, Hakomori S (2001). Binding specificity of siglec7 to disialogangliosides of renal cell carcinoma: possible role of disialogangliosides in tumor progression. FEBS Lett..

[39] Kawasaki Y (2010). Ganglioside DSGb5, preferred ligand for Siglec-7, inhibits NK cell cytotoxicity against renal cell carcinoma cells. Glycobiology.

[40] Saito S (2005). RM2 antigen (beta1,4-GalNAc-disialyl Lc4) as a new marker for prostate cancer. Int. J. Cancer..

[41] Chuang H-Y (2013). Synthesis and vaccine evaluation of the tumor-associated carbohydrate antigen RM2 from prostate cancer. J. Am. Chem. Soc..

[42] Abe A (1995). Structural and stereochemical studies of potent inhibitors of glucosylceramide synthase and tumor cell growth. J. Lipid. Res..

[43] Furukawa K (1985). Analysis of the specificity of five murine anti-blood group A monoclonal antibodies, including one that identifies type 3 and type 4 A determinants. Biochemistry.

[44] Kameyama A, Ishida H, Kiso M, Hasegawa A (1990). Stereoselective synthesis of sialyl-lactotetraosylceramide and sialylneolactotetraosylceramide. Carbohydr. Res..

