# Author Correction: Roles of GalNAc-disialyl Lactotetraosyl Antigens in Renal Cancer Cells

**DOI:** 10.1038/s41598-020-63112-6

**Published:** 2020-04-09

**Authors:** Akiko Tsuchida, Motohiro Senda, Akihiro Ito, Seiichi Saito, Makoto Kiso, Takayuki Ando, Anne Harduin-Lepers, Akio Matsuda, Keiko Furukawa, Koichi Furukawa

**Affiliations:** 1 Laboratory of Glyco-Bioengineering, The Noguchi Institute, Itabashi, 173-0003 Japan; 2 Department of Biochemistry II, Nagoya University Graduate School of Medicine, Nagoya, 466-8550 Japan; 3 Department of Urology, Nagoya University School of Medicine, Nagoya, 466-8550 Japan; 4 Department of Urology, Tohoku University School of Medicine, Sendai, 980-8574 Japan; 5 Department of Urology, University of Ryukyus School of Medicine, Nishihara-cho, 903-0215 Okinawa Japan; 6 Facalty of Applied Biological Sciences, Gifu University, Gifu, 501- 1193 Japan; 7 Department of Drug and Food Science, Shizuoka Institute of Environment and Hygiene, Shizuoka, 420-8637 Japan; 8 Unité de Glycobiologie Structurale et Fonctionnelle, Université Lille Nord de France, Villeneuve d’Ascq, 59655 France; 9 Department of Biomedical Sciences, Chubu University College of Life and Health Sciences, Kasugai, 487-8501 Japan; 10 Department of Lifelong Sports and Health Sciences, Chubu University College of Life and Health Sciences, Kasugai, 487-8501 Japan

Correction to: *Scientific Reports* 10.1038/s41598-018-25521-6, published online 04 May 2018

This Article contains errors.

The cell line used, VMRC-RCZ, was mistakenly referred to as VMRC-RCW throughout the manuscript.

The text under the subheading “Establishment of GalNAc-DSLc4-expressing clones from a renal cancer cell line with transfection of B4GalNAc-T2 cDNA”,

“We also investigated the thin layer chromatography (TLC) pattern of glycolipids from the transfectants and control cells (Supplemental Figure S1).”

should read:

“In the same way, we established the stable transfectants from TUHR14TKB cells, and also investigated the thin layer chromatography (TLC) pattern of glycolipids from the transfectants and control cells (Supplemental Figure S1).”

In addition, in Supplementary Figure S1, the lanes were incorrectly labelled. The correct Supplemental Figure S1 appears below, as Figure 1.

**Figure 1:**
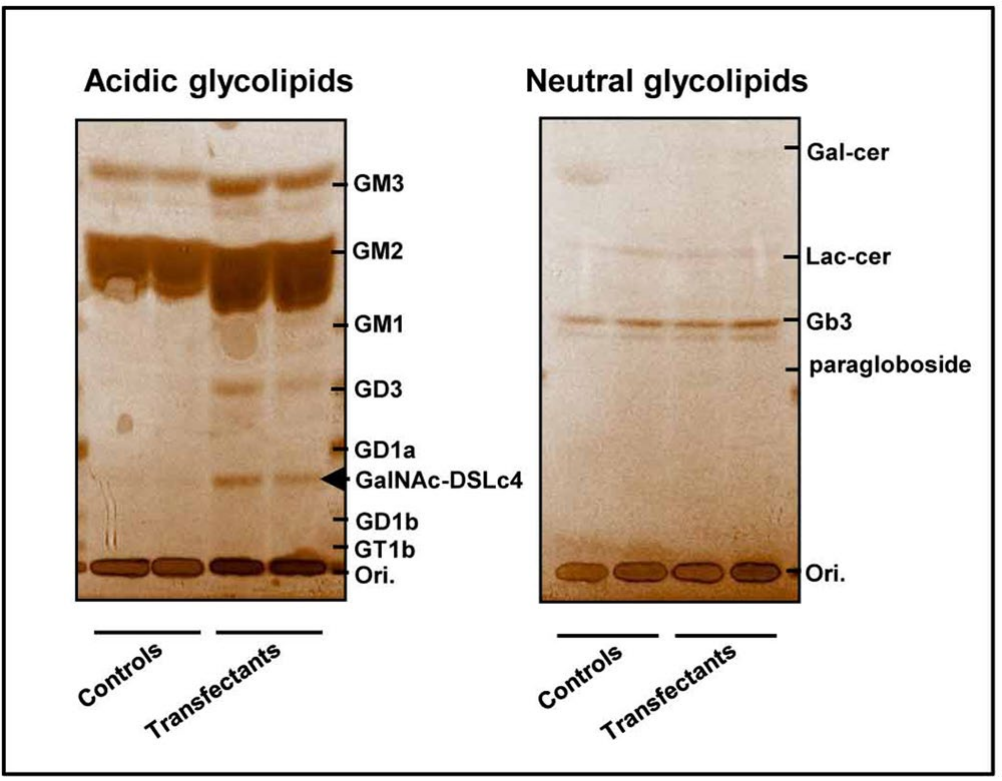
Establishment of B4GalNAc-T2 stable transfectants and neo-expression of GalNAc-DSLc4 in the stable transfectants. TLC of glycolipids extracted from the transfectant cells and control cells. The extracted glycolipids were separated by DEAE-sephadex ion-exchange column chromatography and a C_18_ Sep-Pak cartridge (Waters, Milford, MA). The products were analyzed by TLC with a solvent system of chloroform/methanol/0.2% CaCl_2_ (53:40:7), and detected with orcinol reagent.

Figures 2C was misassembled during the preparation of the manuscript. A new panel c has been added, presenting the adhesion of TUHR14TKB clones onto a surface coated with LN. The corrected Figure 2 appears below, as Figure 2.

**Figure 2:**
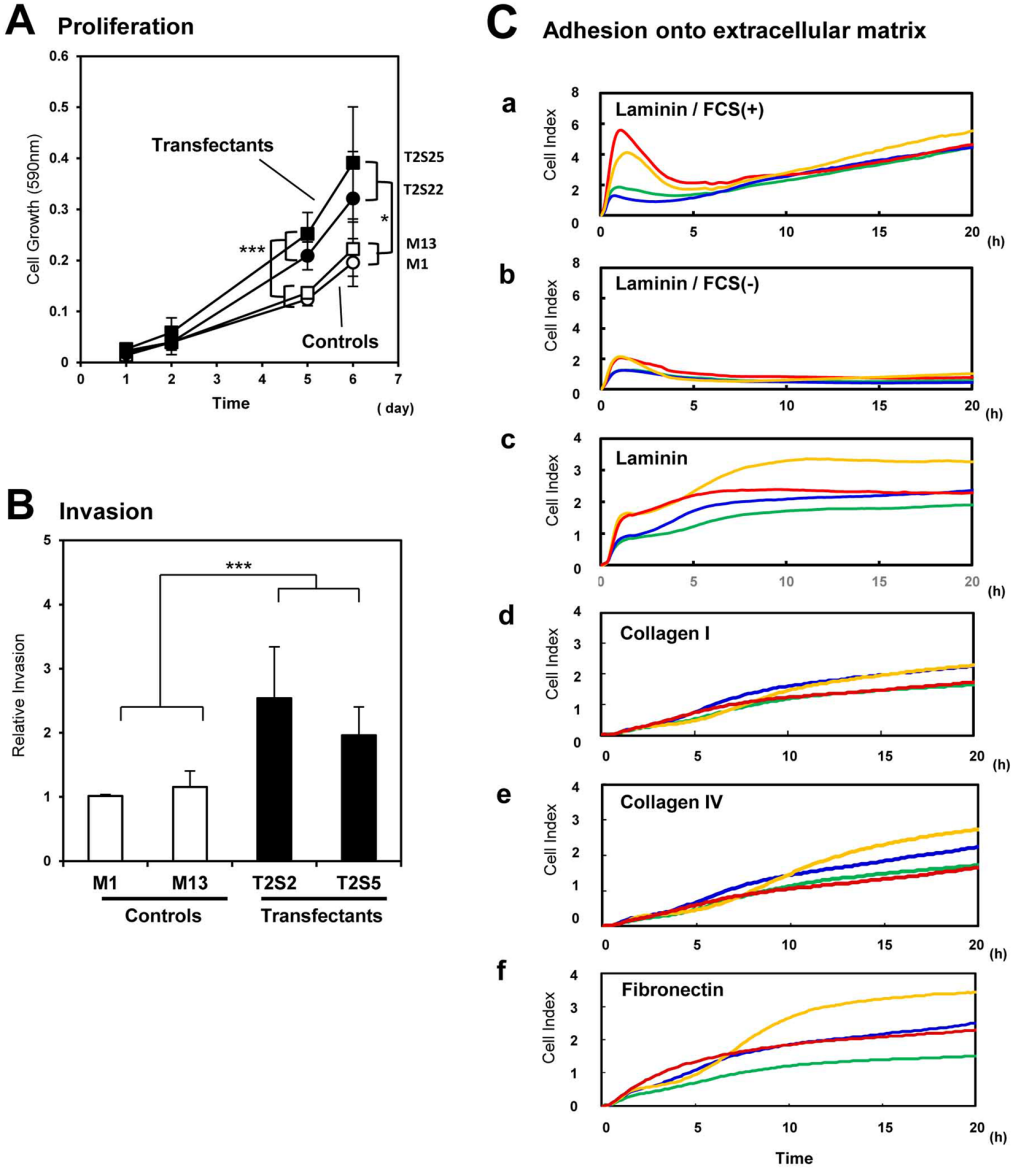
Malignant phenotypes of the B4GalNAc-T2 gene transfectant cells. (**A**) Effects of GalNAc-DSLc4 expression on the cell proliferation. Two transfectants and two vector controls (2.5 × 10^3^ cells/well) were seeded in 48-well plates in serum-containing medium and cultured for 6 days. The absorbance (590 nm) was measured on day 1, 2, 5, and 6. Data are means of three independent experiments. (**B**) Invasion activity of two transfectants and two vector controls. Cell invasion was analyzed by Boyden chamber invasion assay by counting the cell number on the reverse side of the filter. Data are means of three independent experiments. *Bars* indicate mean ± S.D. (n = 3). *, *P* < 0.05, **, *P* < 0.01,***, *P* < 0.005. (**C**) Dynamic monitoring of cell adhesion to LN, CL type I, CL type IV, or FN-coated surfaces. GalNAc-DSLc4-expressing cells and control cells were seeded in the wells of 96-well e-plate at 2.5 × 10^4^ cells/well with FCS, and cell attachment and spreading were monitored by RT-CES system. The e-plates were pre-coated with LN (*a, b, c*), CL type I (*d*), CL type IV (*e*), or FN (*f*). VMRC-RCZ transfectants were used in a and b, and THUR14TKB transfectants were used in c-f. *Red* and *yellow* lines mean transfectant cells, and *green* and *blue* lines mean control cells.

In addition, the text under the subheading “Effects of GalNAc-DSLc4 expression on cell proliferation, invasion and adhesion”,

“Transfectant cells adhered to LN more strongly than control cells in the presence of fetal calf serum (FCS) (Fig. 2C(a)). Adhesion activity of both transfectant cells and control cells for LN were lower under FCS-free conditions than in the presence of FCS (Fig. 2C(b)). For CL type I, CL type VI or FN, the adhesion intensity was very low in either transfectant cells or control cells, and no significant difference was found between them (Fig. 2C(c-e).”

should read:

“Transfectant cells adhered to LN more strongly than control cells in the presence of fetal calf serum (FCS) (Figure 2C(a)). Adhesion activity of both transfectant cells and control cells for LN were lower under FCS-free conditions than in the presence of FCS (Figure 2C(b)). Transfectants derived from THUR14TKB cells also showed similar results (Figure 2C(c)). For CL type I, CL type VI or FN, the adhesion intensity was very low in either transfectant cells or control cells, and no significant difference was found between them (Figure 2C(d, e, f)).”

In Figure 3B, an incorrect blot was used for total-Akt, and the times scales were wrong. The correct Figure 3B, and the associated full blots, appear below as Figures 3 and 4 respectively.

**Figure 3:**
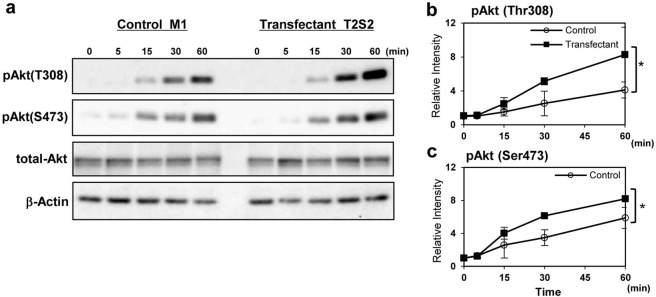
Integrin-ILK-Akt signaling was enhanced in GalNAc-DSLc4-expressing cells. (**A**). Phosphorylation of Akt during treatment with FCS in control cells and GalNAc-DSLc4 expressing cells was examined. Cells were prepared as described in “Materials and Methods”, and cell suspension (4 × 10^5^ cells) were added to plates, and incubated for 0, 10, 30, 60, or 120 min. After incubation, cells were lysed and used for immunoblotting using anti-phospho-Akt (Thr308), anti-phospho-Akt (Ser473), or anti-total Akt antibodies. Bands in autofluorograms (*a*) were quantified by a scanner, and the relative intensities of the bands were plotted after correction with total Akt bands (*b* and *c*). (**B**) Phosphorylation of Akt during adhesion to LN in GalNAc-DSLc4-expressing cells was examined. Cells (4 × 10^5^) were added to pre-coated plates with LN, and incubated for 0, 5, 15, 30, or 60 min. After incubation, cells were lysed and used for immunoblotting using anti-phospho-Akt (Thr308), anti-phospho-Akt (Ser473), or anti-total Akt antibodies. Bands in autofluorograms (*a*) were quantified by a scanner, and the relative intensities of the bands were plotted after correction with total Akt bands (*b* and *c*). *Bars* indicate mean ± S.D. (n = 3). *, *P* < 0.05, **, *P* < 0.01, ***, *P* < 0.005. All cropped blots were run under the same experimental condition. The full-length blots are included in Supplementary Fig.S7 respectively.

**Figure 4:**
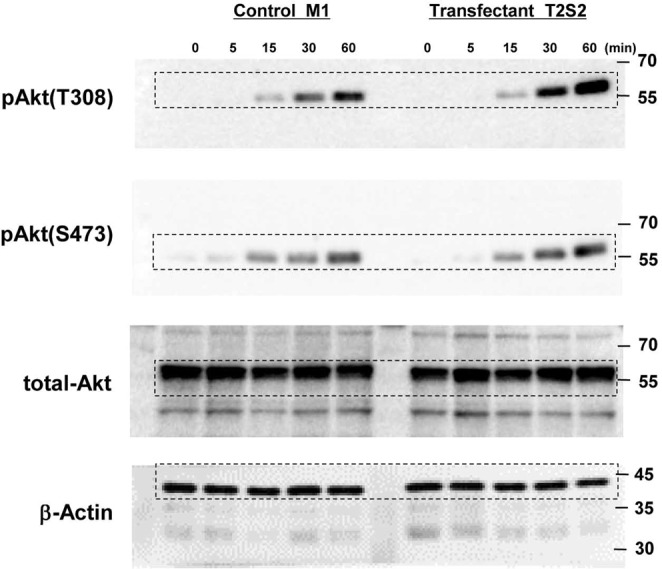
Full-length blots for Figures 3Aa, 3B, 4A, 4Ba, 5A, and 6A.

The text under the subheading “Increased phosphorylation of Akt in the transfectant cells during adhesion to LN”,

“To investigate integrin signaling triggered by cell adhesion to LN, we analyzed phosphorylation of Akt in the transfectant and control cells. After serum starvation and rotation using a tube rotator, cells were plated in dishes pre-coated with LN under FCS (+) condition, and incubated at 37 °C for 0, 15, 30, 60, and 120 min (Fig. 3B). After incubation, cells were lysed and the lysates were immunoblotted using anti-pAkt antibodies. Notably, in the case of cell adhesion to LN, pAkt (Ser473) was activated from 30 min and pAkt (Thr308) was more strongly activated at 120 min in the transfectant cells.”

should read:

“To investigate integrin signaling triggered by cell adhesion to LN, we analyzed phosphorylation of Akt in the transfectant and control cells. After serum starvation and rotation using a tube rotator, cells were plated in dishes pre-coated with LN under FCS (+) condition, and incubated at 37 °C for 0, 5, 15, 30, and 60 min (Figure 3B). After incubation, cells were lysed and the lysates were immunoblotted using anti-pAkt antibodies. Notably, in the case of cell adhesion to LN, pAkt (Ser473) was activated from 15 min and pAkt (Thr308) was more strongly activated at 60 min in the transfectant cells.”

These changes do not affect the conclusions of the Article.

