# Supplementary material for: Roles of GalNAc-disialyl Lactotetraosyl Antigens in Renal Cancer Cells
Source: Sci Rep. 2018 May 4;8:7017. doi: 10.1038/s41598-018-25521-6 (PMC5935701; doi:10.1038/s41598-018-25521-6)

Roles of GalNAc-disialyl Lactotetraosyl Antigens in Renal Cancer Cells

Akiko Tsuchida[1,2], Motohiro Senda[2,3], Akihiro Ito[4], Seiichi Saito[5], Makoto Kiso[6], Takayuki Ando[7], Anne Harduin-Lepers[8], Akio Matsuda[1], Keiko Furukawa[9], Koichi Furukawa[10,*]

[1]Laboratory of Glyco-Bioengineering, The Noguchi Institute, Itabashi, 173-0003, Japan [2]Department of Biochemistry II, Nagoya University Graduate School of Medicine, Nagoya, 466-8550, Japan, [3]Department of Urology, Nagoya University School of Medicine, Nagoya, 466-8550, Japan, [4]Department of Urology, Tohoku University School of Medicine, Sendai, 980-8574, Japan, [5]Department of Urology, University of Ryukyus School of Medicine, Nishihara-cho, 903-0215, Okinawa, [6]Facalty of Applied Biological Sciences, Gifu University, Gifu, 501-1193, Japan, [7]Department of Drug and Food Science, Shizuoka Institute of Environment and Hygiene, Shizuoka, 420-8637, Japan [8] Unité de Glycobiologie Structurale et Fonctionnelle, Université Lille Nord de France, Villeneuve d'Ascq, 59655, France, [9]Department of Biomedical Sciences, Chubu University College of Life and Health Sciences, Kasugai, 487-8501, Japan, [10]Department of Lifelong Sports and Health Sciences, Chubu University College of Life and Health Sciences, Kasugai, 487-8501, Japan

[*] koichi@isc.chubu.ac.jp

Akiko Tsuchida Ph.D
Laboratory of Glyco-Bioengineering,
The Noguchi Institute
1-9-7, Kaga, Itabashi-Ku, Tokyo 173-0003 JAPAN
E-mail: akikots@noguchi.or.jp
Tel: +81-3-5248-6025
Fax: +81-3-5944-3220

[*]Corresponding Author:
Koichi Furukawa MD, PhD
Department of Lifelong Sports and Health Sciences, Chubu University College of Life and Health Sciences,
1200, Matsumoto-Cho, Kasugai City, Aichi 487-8501 JAPAN
E-mail: koichi@isc.chubu.ac.jp
Tel: +81-568-51-9512
Fax: +81-568-51-9512

Supplementary Information

Supplementary methods

Construction of expression vectors

The expression vector pcDNA3.1 (+)–B4GalNAc-T1 was prepared by inserting a *Xba*I and *Xho*I fragment from pMIKneo–B4GalNAc-T1 into *Xba*I and *Xho*I sites of pcDNA3.1(+) vector. The expression vector pcDNA3.1 (+)–B4GalNAc-T2 was prepared by inserting a *Xba*I and *Xho*I fragment from human kidney library into *Xba*I and *Xho*I sites of pcDNA3.1(+) vector. The expression vector pcDNA3.1 (+)–B4GalNAc-T3 and pcDNA3.1–B4GalNAc-T4 were prepared by inserting a *Xba*I and *Xho*I fragment from human brain library into *Xba*I and *Xho*I sites of pcDNA3.1(+) vector. The expression vector pcDNA3.1 (+)–CSGalNAc-T1 and pcDNA3.1–CSGalNAc-T2 were prepared by inserting a *Xba*I and *Xho*I fragment from human thyroid library and human small intestine library, respectively, into *Xba*I and *Xho*I sites of pcDNA3.1(+) vector.

Preparation of membrane fraction

L cells (3×10^6) were plated in 10-cm dishes at least 48 h prior to transfection. Cells were transiently transfected with an expression plasmid (4 μg) by Lipofectamine™ 2000 (*Thermo Fisher Scientific*) according to the manufacturer's instructions. After 48 h of culture in D-MEM containing 7.5% FCS, cells were harvested by trypsinization. Cells were pelleted, washed with phosphate-buffered saline (PBS), and lysed in ice-cold PBS containing 1 mM phenylmethylsulfonyl fluoride (PMSF) using a nitrogen cavitation apparatus (Parr Instrument Co., Moline, IL) at 400 p.s.i. for 30 min. Nuclei were removed by low speed centrifugation, and supernatant was centrifuged at 100,000 × *g* for 1 h at 4 °C. The pellet was resuspended in ice-cold 100 mM sodium cacodylate buffer, pH 7.2 and used as an enzyme source for *N*-acetylgalactosaminyltransferase assay as described below.

N-acetylgalactosaminyltransferase assay

The *N*-acetylgalactosaminyltransferase assay was performed in a mixture containing 100 mM sodium cacodylate buffer, pH 7.2, 10 mM $MnCl_2$, 0.3% Triton X-100, 1 mM CDP-choline (Sigma), 0.1 mM UDP-GalNAc (Sigma), 25,000 dpm/nmol UDP-[^{14}C]GalNAc (Amersham Pharmacia Biotech), 50 μg of a membrane fraction, and 5 μg of disialyl Lc4 as an acceptors in total volume of 50 μl. The reaction mixture was incubated at 37 °C for 12 h. The products were isolated using a C_{18} Sep-Pak cartridge (Waters, Milford, MA) and analyzed by TLC with a solvent system of chloroform/methanol/0.2% $CaCl_2$ (55:45:10). The radioactivity on each plate was visualized with a BAS 2000 image analyzer (Fuji Film, Tokyo, Japan). For acceptors, Lc4 (lactotetraosyl ceramide : Galβ1,3GlcNAcβ1,3Galβ1,4Glcβ1-Cer),

SLc4 (sialyl lactotetraosylceramide : NeuAcα2,3Galβ1,3GlcNAcβ1,3Galβ1,4Glcβ1-Cer), nSLc4 (sialyl neolactotetraosylceramide : NeuAcα2,3Galβ1,4GlcNAcβ1,3Galβ1,4Glcβ1-Cer), DSLc4 (disialyl lactotetraosylceramide : NeuAcα2,3Galβ1,3(NeuAcα2,6)GlcNAcβ1,3Galβ1,4Glcβ1-Cer) were prepared as described[44].

Transfection for flow cytometric analysis

The THUR14TKB cells in 6-cm dish (Falcon) were transiently transfected with pcDNA3.1, pcDNA3.1–B4GalNAc-T1, pcDNA3.1–B4GalNAc-T2, pcDNA3.1–B4GalNAc-T3, pcDNA3.1–B4GalNAc-T4, pcDNA3.1–CSGalNAc-T1 and pcDNA3.1–CSGalNAc-T2 by Lipofectamine™ 2000 and cultured for 48 h in D-MEM containing 7.5% FCS before observation. The transfected cells were trypsinized and washed twice with PBS, then used for flow cytometric analysis using mAb RM2.

Glycolipid extraction and TLC[41]

Briefly, glycolipids were extracted from ~400 μl of packed cells using chloroform/methanol (2:1, 1:1, and 1:2), sequentially. TLC was performed with high-performance TLC plates (Merk, Darmstadt, Germany) using a solvent system of chloroform/methanol/0.2% $CaCl_2$aq. (60:35:8) and sprayed by orcinol or primulin. For standards, purified mixed gangliosides were purchased from Matraya LLC. (Pleasant Gap, PA).

In vitro invasion assay

Invasion assay were performed using BioCoat™ Matrigel™ Invasion Chamber (Corning). In brief, cells (2.5×10^4 cells/well) were added to serum-free medium in the upper chamber and incubated for 18 h at 37 °C. Subsequently, the cells on the upper surface of filter were removed completely by wiping with a cotton swab. The filter were fixed in ethanol and stained with Giemsa (Wako Pure Chemical). The number of invaded cells was counted. Assays were carried out in triplicate.

Preparation of GEM/raft fractions

In brief, cells (2-2.5 × 10^7) were plated in 15-cm culture dishes, cultured up to 90% confluency, and then two dishes of cells were used for each preparation. After washing twice by ice-cold PBS, the cells were lysed with 1 ml of MNE/Triton X-100 buffer (1% Triton X-100, 25 mM MES, pH 6.5, 150 mM NaCl, 5 mM EDTA, 1 mM Na_3VO_4, 1 mM PMSF, 1 μg/ml of aprotinin) and then Dounce-homogenized 10 times with a Digital Homogenizer™ (AS ONE, Osaka, Japan). Insoluble materials were removed by centrifugations at 15,000 × g for 10 min. The lysates were placed on the bottom of Ultra-Clear™ centrifuge tubes (Beckman Instruments) and mixed with an equal volume of 80% sucrose in MNE buffer, and stepwise gradient was prepared by overlaying 30% sucrose in MNE followed by a final layer of 5%

sucrose in MNE. The samples were centrifuged at 200,000 × g in a Beckman SW50.1 rotor (Kent, MI) for 16 h at 4 °C. Fractions of 1 mL were separated from the top of the gradient, and were used for Western immunoblotting.

Immunofluorescence staining

Cells were rotated under serum-free conditions for 30 min, and plated on pre-coated plate with LN in D-MEM including 10% FCS, and incubated for 0-60 min. Cells were fixed in 4% paraformaldehyde for 10 min at room temperature. After being washed with PBS, nonspecific binding was blocked with 2.5% normal donkey serum and 5% normal goat serum in PBS for 30 min at room temperature. Cells were incubated with biotin-conjugated anti-human CD29 antibody or mAb RM2 in PBS for 45 min at room temperature, then with streptavidin-Alexa 488 or anti-mouse IgM-Alexa 594, respectively, in PBS for 30 min at room temperature. The resulting staining patterns were imaged using a confocal microscope (Fluoview FV10i-DOC, Olympus, Tokyo, Japan).

Supplementary Figure Legends

Supplemental Fig. S1

Establishment of B4GalNAc-T2 stable transfectants and neo-expression of GalNAc-DSLc4 in the stable transfectants

TLC of glycolipids extracted from the transfectant cells and control cells. The extracted glycolipids were separated by DEAE-sephadex ion-exchange column chromatography and a C_{18} Sep-Pak cartridge (Waters, Milford, MA). The products were analyzed by TLC with a solvent system of chloroform/methanol/0.2% $CaCl_2$ (53:40:7), and detected with orcinol reagent.

Supplemental Fig. S2

A, Phosphorylation of Akt during treatment with 50 ng/ml EGF in control cells and GalNAc-DSLc4-expressing cells was examined. Cells were prepared as described in “Materials and Methods”, and cell suspension (4×10^5 cells) were added to plates, and incubated for 0, 15, 30, or 60 min. After incubation, cells were lysed and used for immunoblotting using anti-phospho-EGFR (Tyr1068), anti-phospho-ERK (p44/p42), anti-phospho-Akt (Thr308), or anti-phospho-Akt (Ser473) antibodies. Band in autofluorograms (*a*) were quantified by a scanner, and the relative intensities of bands were plotted after correction with β-actin bands. *B*, Phosphorylation of Akt during treatment with 100 ng/ml HGF in control cells and GalNAc-DSLc4 expressing cells was examined. Cells were prepared as described above, and cell suspension (4×10^5 cells) were added to plates, and incubated for 0, 5, 15, 30, or 60 min. After incubation, cells were lysed and used for immunoblotting using anti-phospho-cMet (Tyr1234/1235),

anti-phospho-ERK (p44/p42), anti-ERK, anti-phospho-Akt (Thr308), anti-phospho-Akt (Ser473), or anti-Akt antibodies. Bands in autofluorograms (*a*) were quantified by a scanner, and the relative intensities of the bands were plotted after correction with total Akt bands. *Bars* indicate mean ± S.D. (n=3). *, $P< 0.05$, **, $P< 0.01$, ***, $P< 0.005$.

Supplemental Fig. S3

Spatio-temporal relationships between integrins and GalNAc-DSLc4 during adhesion

A. Transfectant cells were plated on pre-coated plates with laminin after being treated and rotated under serum-free conditions as described in "Materials and Methods". After incubation for 0, 5, 10, 30, 60, and 120 min, cells were fixed with 4% paraformaldehyde. Then cells were stained for integrin β1 (ITGB1, *green*) and GalNAc-DSLc4 (RM2, *red*), and their images were observed using a confocal microscope. *B*. Similar images as shown in *F*, the number in the images correspond to the number in F. *b*, images of the *x-z* axis in a (*purple line*). The *bottom* side in the image is the adhesion site. *c*, images of the *y-z* axis in a (*yellow line*). The *left* side in the image is the adhesion site. In all experiments, it was confirmed that there was no cross-reaction between the individual antigens and non-relevant second reagents. *DIC*, image of differential interference contrast microscope. *Scale bars* indicate 10 μm.

Supplemental Fig. S4

Sialyltransferase assay of ST6GalNAcVI enzyme toward GalNAc-SLc4 as an acceptor

A. Sialyl taransferase assay was performed using ST6GalNAc VI enzyme and GalNAc-sialyl Lc4 as an acceptor. The products were analyzed by TLC. *B*, GalNAc-DSLc4 was not detected in the assay using GalNAc-SLc4 as an acceptor. The results revealed that the precursor of GalNAc-DSLc4 is DSLc4, but not GalNAc-SLc4.

Supplemental Fig. S5

Correlation between the expression levels of GalNAc-T2 isoforms (long form and short form) and the expression levels of GalNAc-DSLc4 in RCCs

B4GalNAc-T2 was not detected in HRPTE (human renal proximal tubule epithelial cells) but often found in renal carcinoma cell lines. The short form was principally expressed in RCCs, suggesting that this isoform correlates with the expression levels of GalNAc-DSLc4 antigen. The glycolipid expression levels were classified into 5 groups based on the percentages of positive cells. +++, 70-100%; ++, 40-70%; +, 10-40%; ±, 5-10%; –, 0-5%.

Supplemental Fig. S6

A schema to show roles of GalNAc-DSLc4 in RCC cells

Expression of GalNAc-DSLc4 in transfectant cells enhances the formation of platforms that function in

membrane signaling and trafficking, leading to the generation of raft “phase”. The enhancement of the function of integrin β1 and growth factor receptors in the raft “phase” might result in enhancement of malignancy.

Supplemental Fig. S7

Full-length blots for Figures 3Aa, 3B, 4A, 4Ba, 5A, and 6A.

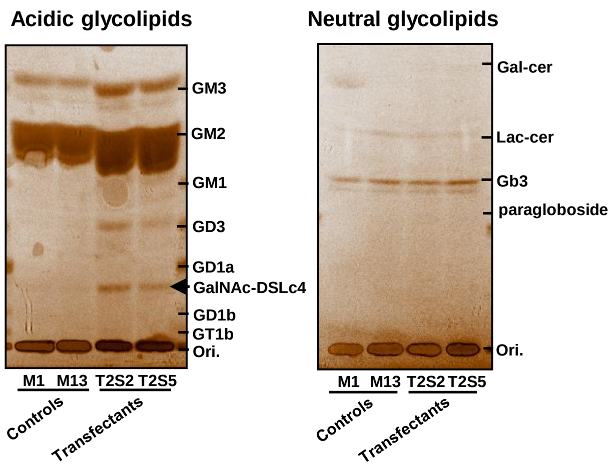

Acidic glycolipids
Neutral glycolipids
GM3
GM2
GM1
GD3
GD1a
GalNAc-DSLc4
GD1b
GT1b
Ori.
M1 M13 T2S2 T2S5
Controls
Transfectants
Gal-cer
Lac-cer
Gb3
paragloboside
Ori.
M1 M13 T2S2 T2S5
Controls
Transfectants

A

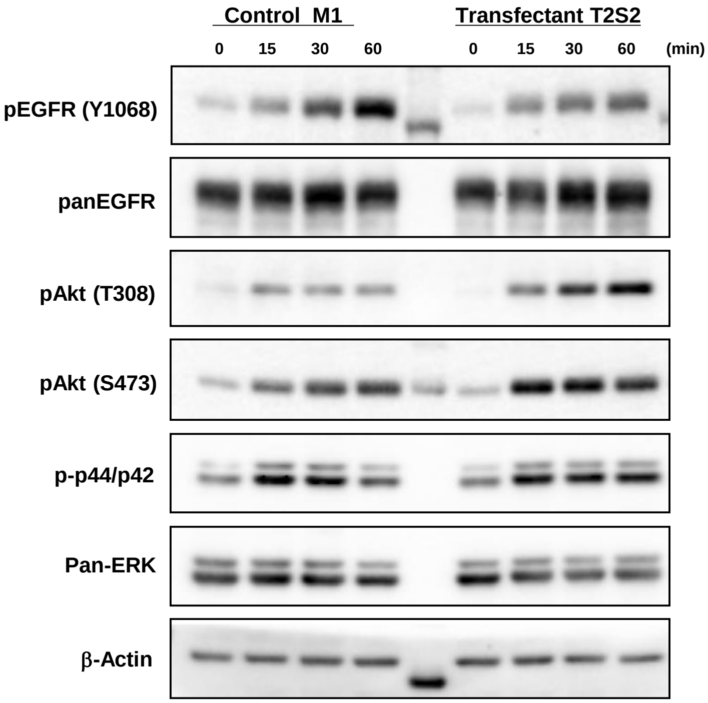
Control M1
Transfectant T2S2
0
15
30
60
0
15
30
60
(min)
pEGFR (Y1068)
panEGFR
pAkt (T308)
pAkt (S473)
p-p44/p42
Pan-ERK
β-Actin

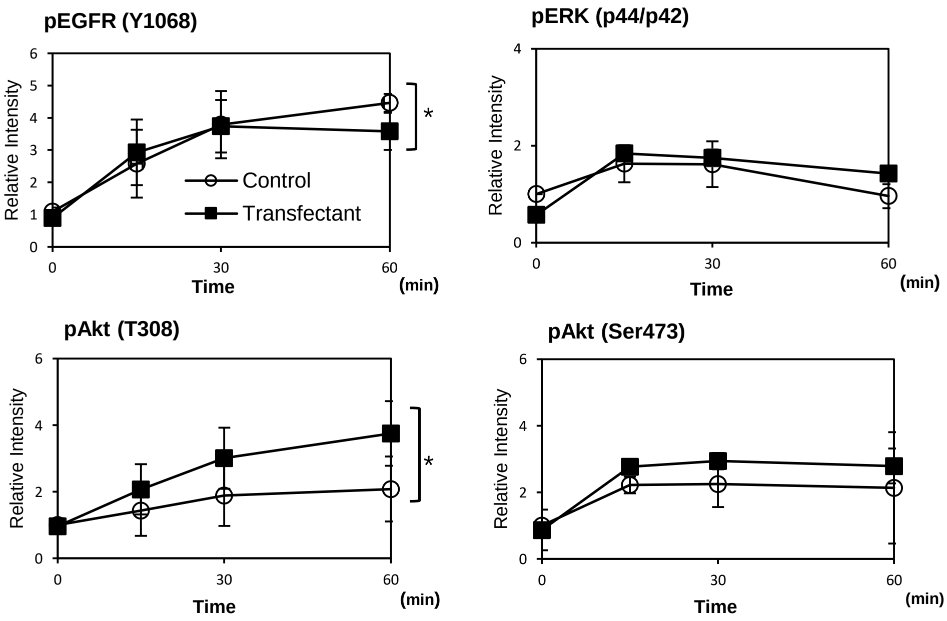
pEGFR (Y1068)
Relative Intensity
Time
(min)
Control
Transfectant
*
pERK (p44/p42)
Relative Intensity
Time
(min)
pAkt (T308)
Relative Intensity
Time
(min)
*
pAkt (Ser473)
Relative Intensity
Time
(min)

B

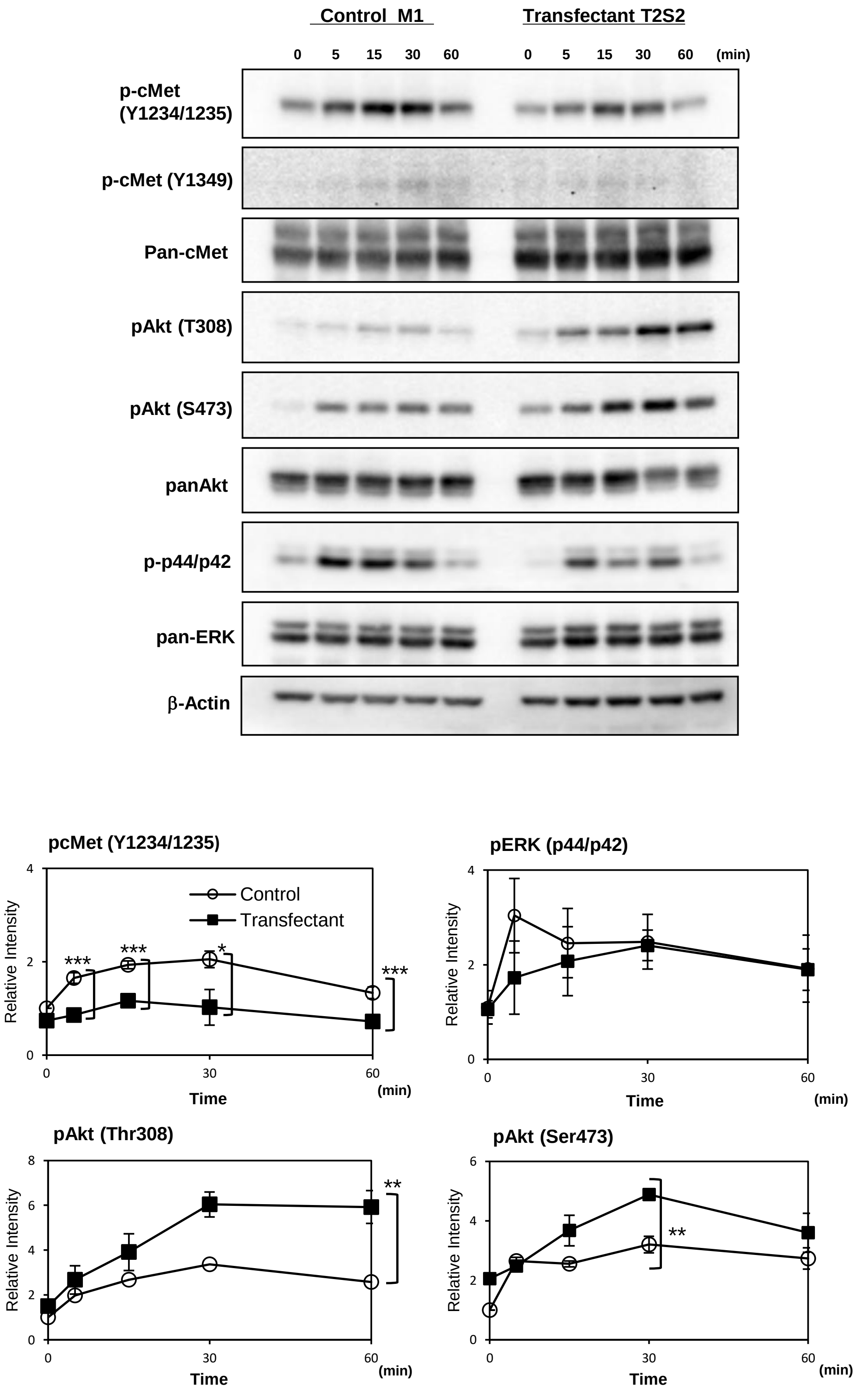

Control M1
Transfectant T2S2
0 5 15 30 60 0 5 15 30 60 (min)
p-cMet (Y1234/1235)
p-cMet (Y1349)
Pan-cMet
pAkt (T308)
pAkt (S473)
panAkt
p-p44/p42
pan-ERK
β-Actin
pcMet (Y1234/1235)
Control
Transfectant
***
***
*
***
Relative Intensity
Time
(min)
pERK (p44/p42)
pAkt (Thr308)
**
pAkt (Ser473)
**

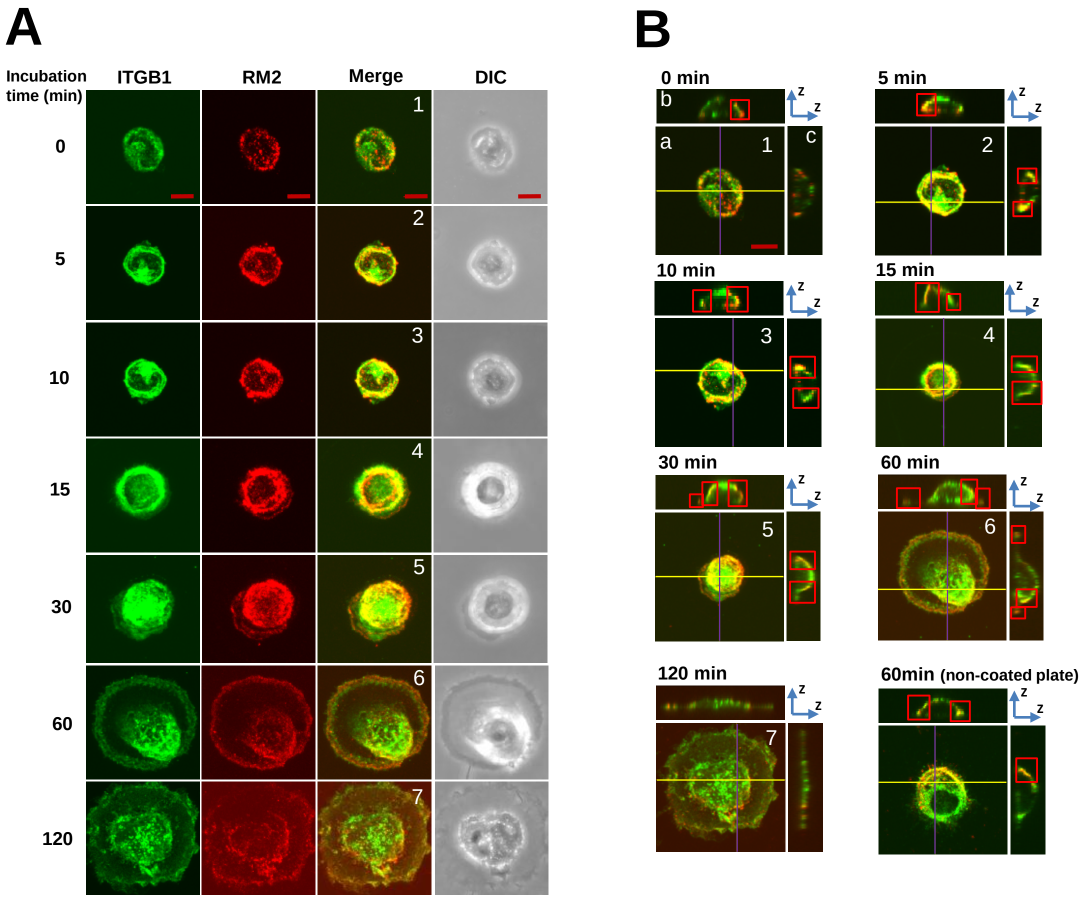
A
Incubation time (min)
ITGB1
RM2
Merge
DIC
0
5
10
15
30
60
120
1
2
3
4
5
6
7
B
0 min
5 min
10 min
15 min
30 min
60 min
120 min
60min (non-coated plate)
a
b
c
z

A **Sialyltransferase assay toward GalNAc-SLc4 as an acceptor**

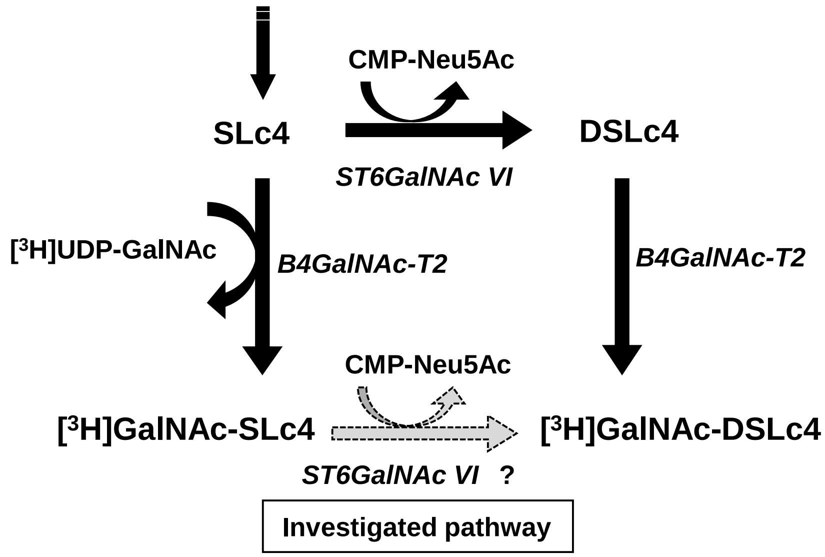

B **TLC of enzyme products**

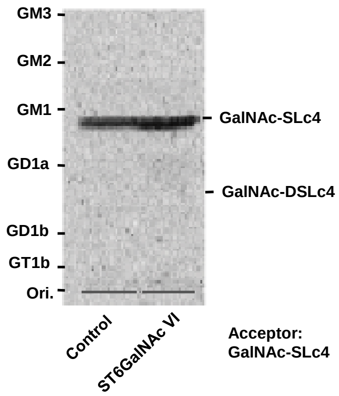

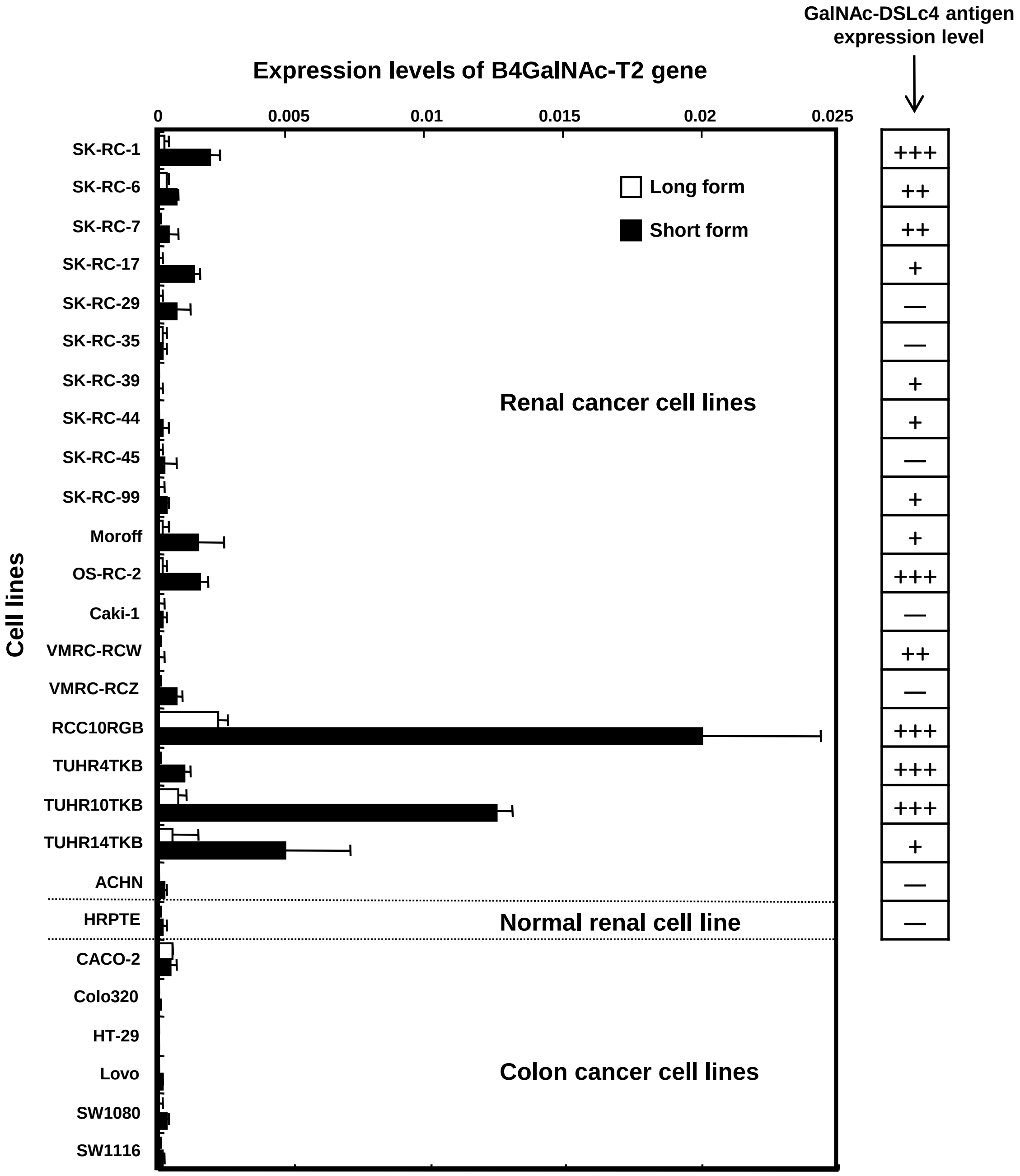

GalNAc-DSLc4 antigen expression level
Expression levels of B4GalNAc-T2 gene
0
0.005
0.01
0.015
0.02
0.025
Long form
Short form
Cell lines
SK-RC-1
SK-RC-6
SK-RC-7
SK-RC-17
SK-RC-29
SK-RC-35
SK-RC-39
SK-RC-44
SK-RC-45
SK-RC-99
Moroff
OS-RC-2
Caki-1
VMRC-RCW
VMRC-RCZ
RCC10RGB
TUHR4TKB
TUHR10TKB
TUHR14TKB
ACHN
HRPTE
CACO-2
Colo320
HT-29
Lovo
SW1080
SW1116
Renal cancer cell lines
Normal renal cell line
Colon cancer cell lines
+++
++
++
+
—
—
+
+
—
+
+
+++
—
++
—
+++
+++
+++
+
—
—

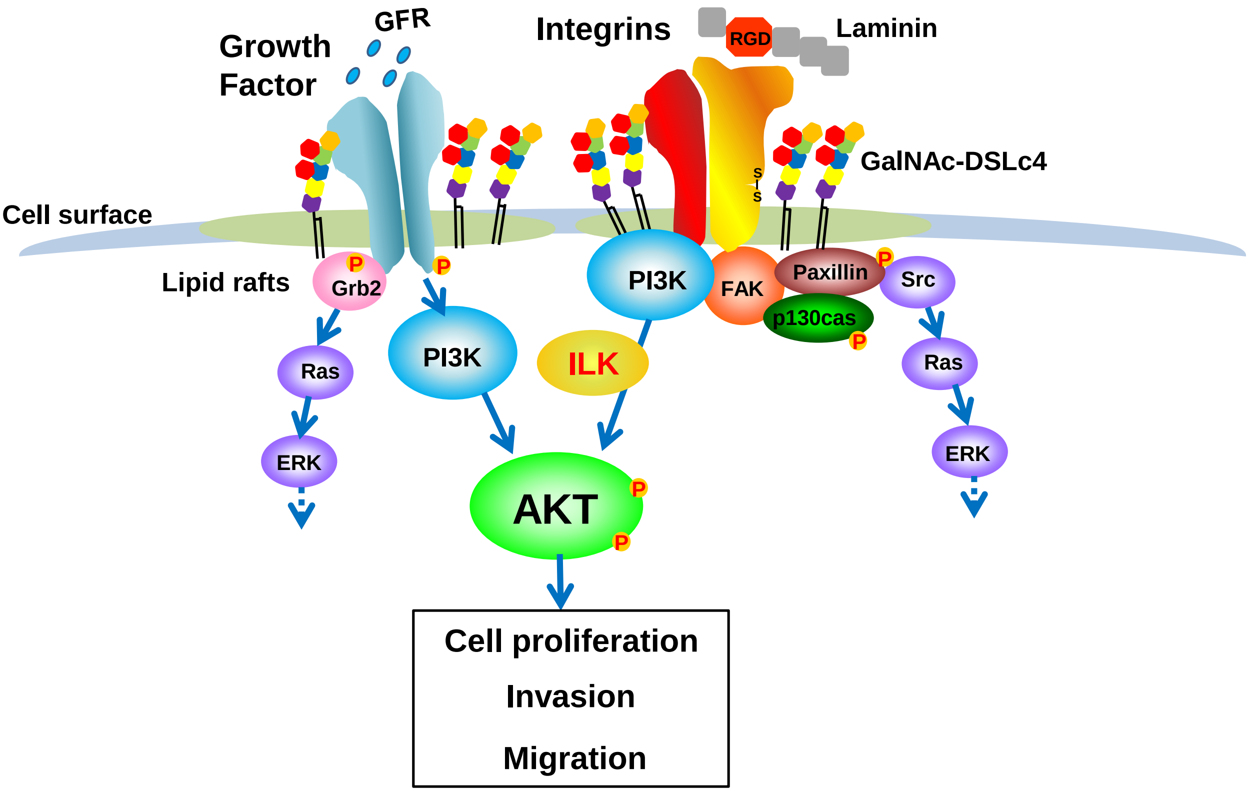

Growth Factor
GFR
Integrins
RGD
Laminin
GalNAc-DSLc4
Cell surface
Lipid rafts
P
Grb2
P
PI3K
FAK
Paxillin
P
Src
p130cas
P
Ras
PI3K
ILK
Ras
ERK
ERK
AKT
P
P
S
S
Cell proliferation
Invasion
Migration

Relative to Fig.3A

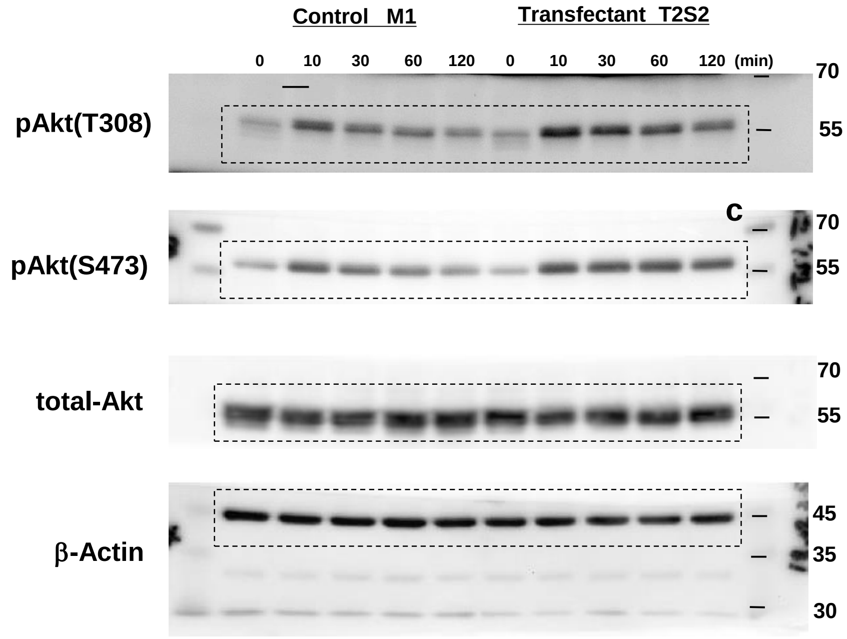

Relative to Fig.3B

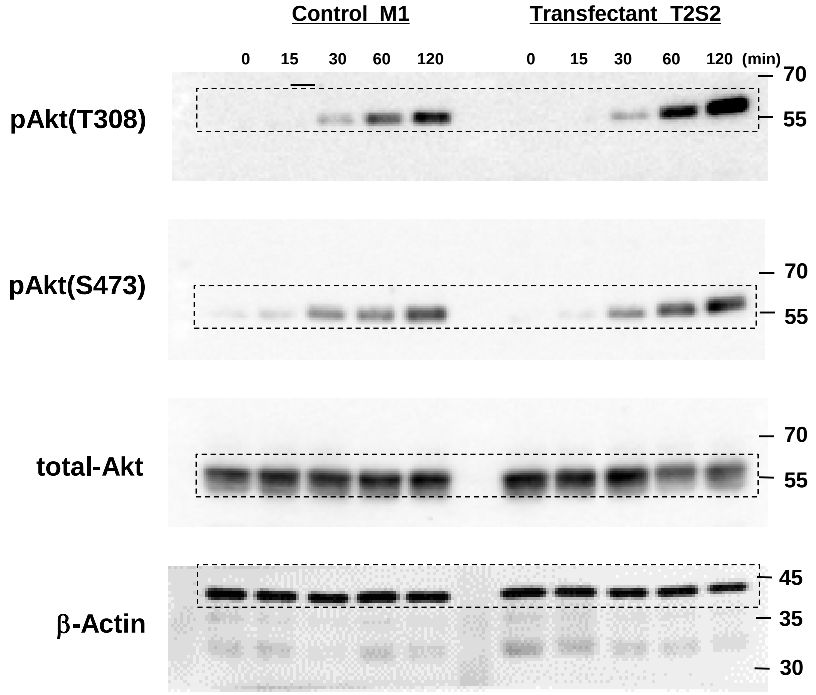

Supplemental Fig. S7

Tsuchida et al.

Relative to Fig.4A

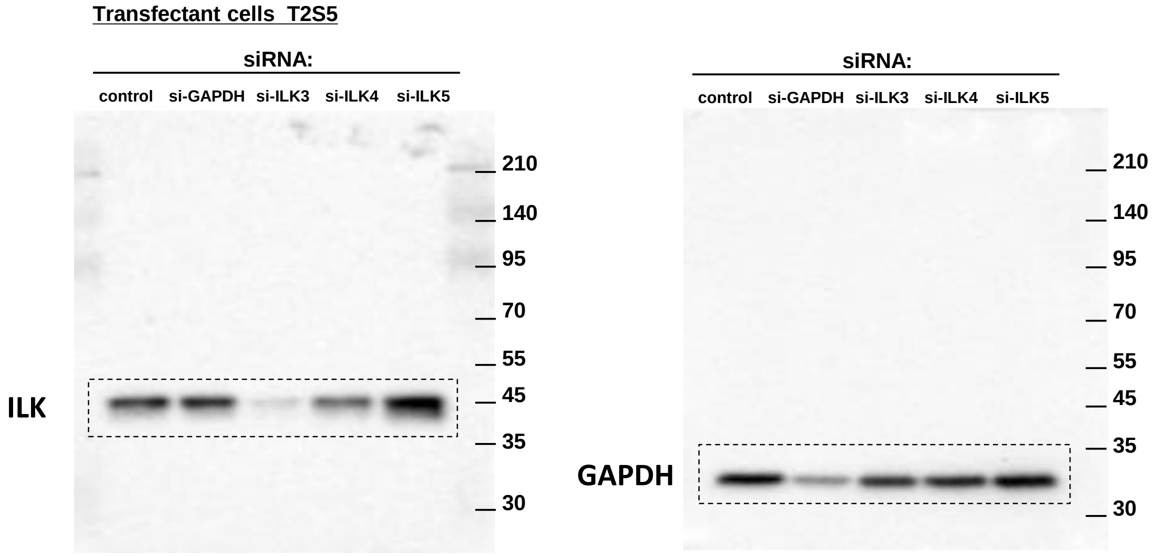

Relative to Fig.4Ba

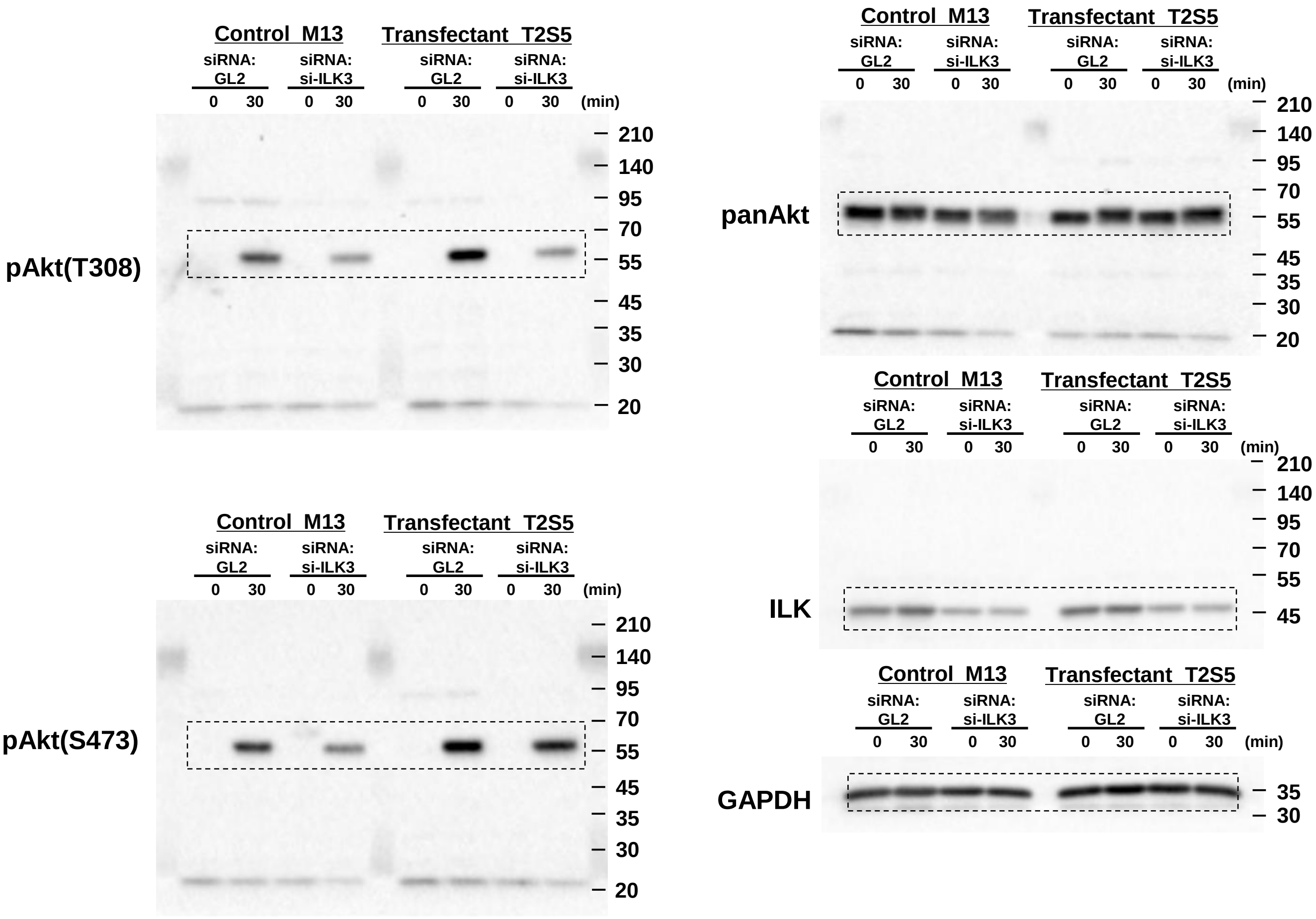

Tsuchida et al.

Supplemental Fig. S7

Relative to Fig.5A

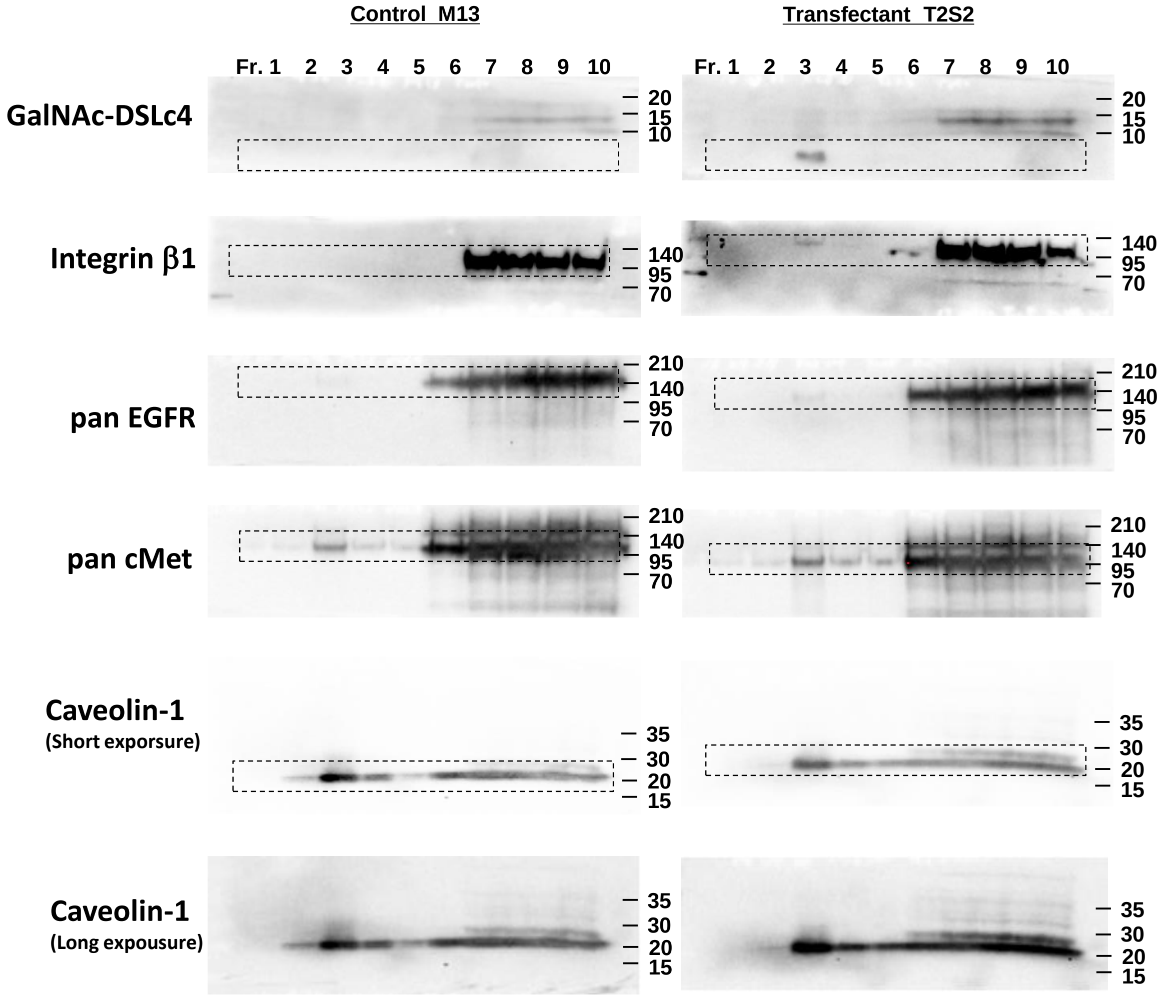

Tsuchida et al.

Supplemental Fig. S7

Relative to Fig.6

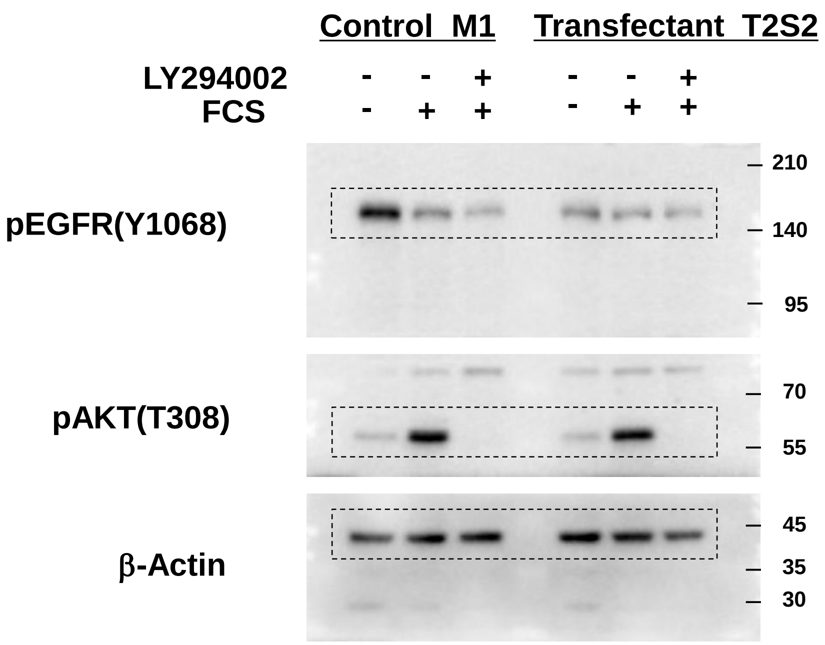

Supplement: Supplementary file 1 — Suplemental Information [file 41598_2018_25521_MOESM1_ESM.pdf]
